# Family characteristics, phylogenetic reconstruction, and potential applications of the plant BAHD acyltransferase family

**DOI:** 10.3389/fpls.2023.1218914

**Published:** 2023-10-05

**Authors:** Donghuan Xu, Zhong Wang, Weibing Zhuang, Tao Wang, Yinfeng Xie

**Affiliations:** ^1^ Co-Innovation Center for Sustainable Forestry in Southern China, College of Life Sciences, Nanjing Forestry University, Nanjing, China; ^2^ Jiangsu Key Laboratory for the Research and Utilization of Plant Resources, Institute of Botany, Jiangsu Province and Chinese Academy of Sciences (Nanjing Botanical Garden Mem. Sun Yat-Sen), Nanjing, China

**Keywords:** BAHD acyltransferase, protein structure, catalytic mechanism, phylogenetic reconstruction, acylation reactions

## Abstract

The BAHD acyltransferase family is a class of proteins in plants that can acylate a variety of primary and specialized secondary metabolites. The typically acylated products have greatly improved stability, lipid solubility, and bioavailability and thus show significant differences in their physicochemical properties and pharmacological activities. Here, we review the protein structure, catalytic mechanism, and phylogenetic reconstruction of plant BAHD acyltransferases to describe their family characteristics, acylation reactions, and the processes of potential functional differentiation. Moreover, the potential applications of the BAHD family in human activities are discussed from the perspectives of improving the quality of economic plants, enhancing the efficacy of medicinal plants, improving plant biomass for use in biofuel, and promoting stress resistance of land plants. This review provides a reference for the research and production of plant BAHD acyltransferases.

## 1 Introduction

Acylation is a common chemical reaction in living organisms that catalyzes a series of oxygenated and nitrogenous compounds to synthesize corresponding ester and amide products (Wang et al., 2022). The BAHD acyltransferase family is a group of proteins that acylate primary and specialized secondary metabolites in plants. Members of this family mainly use acyl-coenzyme A as the acyl donor to produce various volatile lipids, modified anthocyanins, and compounds related to plant resistance to pathogenic microorganisms, thus playing important roles in signal transduction, stress defense, and metabolic homeostasis (Rosa and Neish, 1968; Suzuki et al., 2004b; D’Auria, 2006).

**Figure 1:**
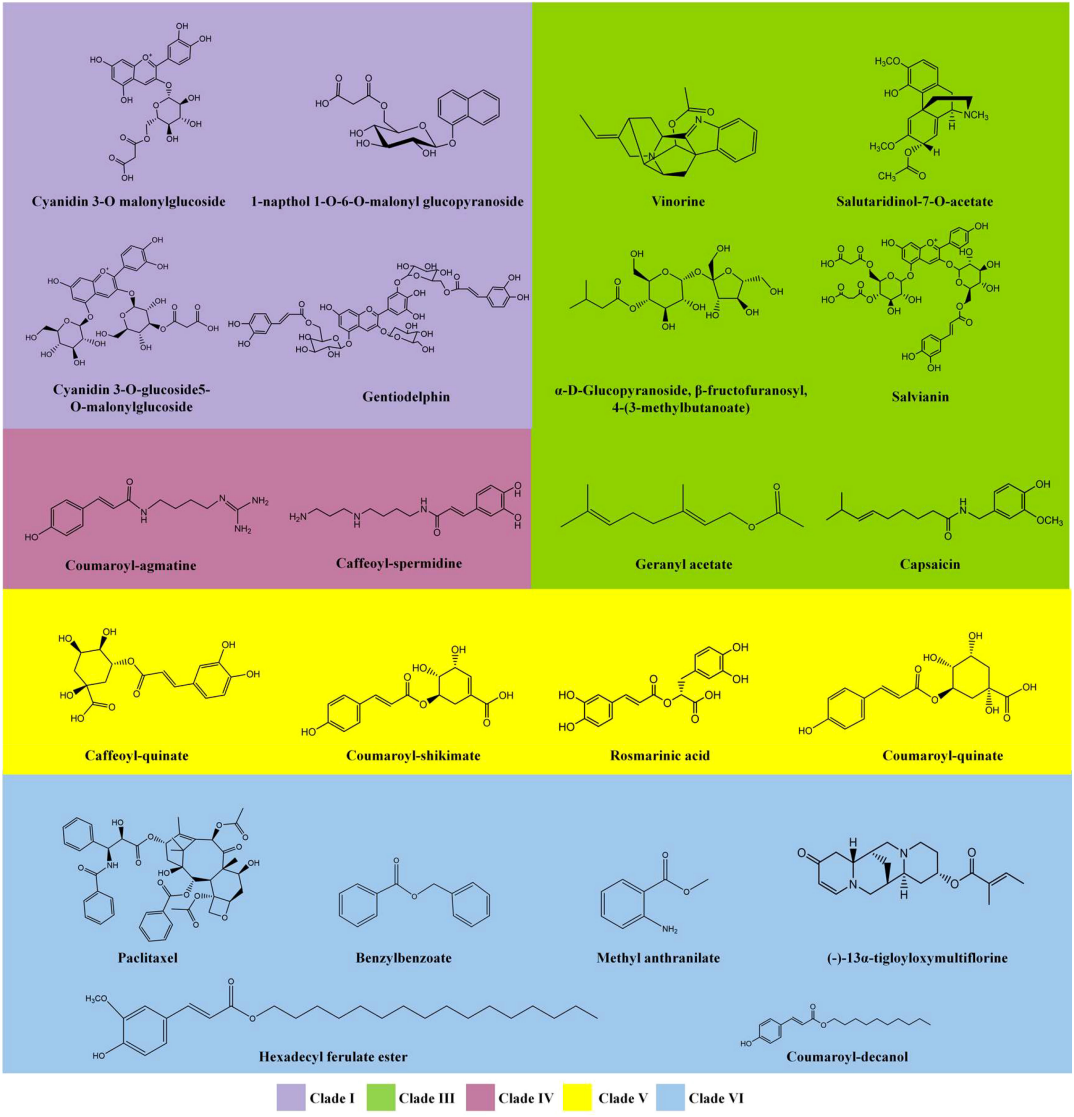
Representative metabolites that are acylated by BAHD family members. Compounds are categorized based on the clade relationships of the respective BAHD family members (**Figure 3**). More structures could be acquired from **Supplemental Table S1**.

The BAHD acyltransferase family was named according to the first letter of each of the first four biochemically characterized enzymes within this family: benzylalcohol O-acetyltransferase (BEAT), anthocyanin O-hydroxycinnamoyltransferase (AHCT), anthranilate N-hydroxycinnamoyl/benzoyltransferase (HCBT), and deacetylvindoline 4-O-acetyltransferase (DAT) (D’Auria, 2006). Members of the BAHD family have been reported in model plants, such as Arabidopsis, Barley, Rice, and Poplar, as well as in important medicinal and economic plants, including Pear, Chinese staff vine (*Celastrus angulatus*), Jasmine, Tea, and Taxus (Aktar et al., 2022; Grienenberger et al., 2009; Zhang and Xu, 2018; Liu et al., 2020; Yamane et al., 2020; Yan et al., 2020; Wang et al., 2021a; Wang et al., 2021b; Zhao et al., 2021; Yuan et al., 2022). These BAHD proteins are involved in the formation of a variety of plant-derived active acylated natural products and their precursors, such as anthocyanins, alkaloids, aromatic alcohols/amines, aliphatic alcohols/amines, terpenoids and sugar detivatives. Based on the clade relationships of BAHD family, a series of representative compounds were shown in **Figure 1**, including cyanidin 3-O malonylglucoside, vinorine (a precursor to vincristine), geranyl acetate, coumaroyl-agmatine, caffeoyl quinic acid, and paclitaxel (taxol). A deeper understanding of these modifications may provide new opportunities for metabolic engineering and synthetic biology of such compounds.

Here, we review plant BAHD acyltransferases and the characteristics, including the protein structure and catalytic mechanisms. We describe the types of acylation reactions mediated by BAHD acyltransferases, as well as the potential functional differentiation, which has been studied by phylogenetic reconstruction. Moreover, the BAHD family has broad potential applications in human activities and four key aspects will be discussed, including improving the quality of economic plants, enhancing the efficacy of medicinal plants, improving plant biomass for use in biofuel, and promoting the stress resistance of land plants. This review provides a theoretical and practical basis for further research on the functions of plant BAHD acyltransferases and their potential applications.

## 2 Characteristics of BAHD acyltransferases

### 2.1 Protein structure

The BAHD members are globular proteins that are mostly localized in the cytosol, while a few are localized in the nucleus, such as *Medicago truncatula* anthocyanin 5-O-glucoside-6’’’-O-malonyltransferase (MtMaT1) (Yu et al., 2008). They have a molecular mass ranging between 48 and 55 kDa, and the average number of amino acids is 445 (Suzuki et al., 2004b). The primary structure of plant BAHDs is varied, and some sequences with different clades only show 10–30% similarity at the amino acid level (Rosa and Neish, 1968); while the similarity within some pairwise comparisons of functionally equivalent members from different species, such as proteins that synthesize benzoic acid methyl esters, is as high as 90% (Nakayama et al., 2003). Despite the varied similarity between BAHD proteins, the typically sequences of all proteins in this family share two conserved motifs: HXXXD and DFGWG (Unno et al., 2007). The HXXXD motif is located near the center of each enzyme, which is essential for catalysis, and is absolutely conserved in BAHD acyltransferases. The DFGWG motif is located near the C-terminal of the protein and might play an important role in the catalytic process and binding of CoA. The DFGWG motif is not entirely conserved and, for example, is DFGFG, DFGWA, or DFGWK in poplars (Liu et al., 2016a). Moreover, the BAHD family members responsible for the synthesis of anthocyanins often contain an additional conserved motif, i.e., YFGNC (Nakayama et al., 2003).

**Figure 2:**
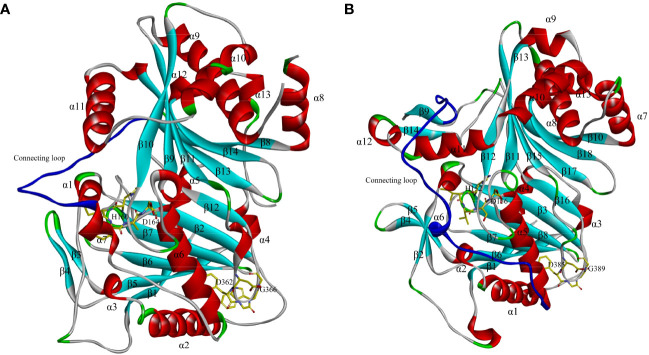
Crystal structures of *Rauvolfia serpentina* vinorine synthase (*Rs*VS) **(A)** and *Hordeum vulgare* agmatine coumaroyltransferase (*Hv*ACT) **(B)**. The structure of *Rs*VS is reproduced according to reference reported by Ma et al. (2005), and the structure of is *Hv*ACT reproduced according to reference reported Yamane et al. (2020).

Despite the sequence differences among BAHD members, their spatial structures are similar (Unno et al., 2007). The first characterized crystal structure of a BAHD member was of *Rauvolfia serpentina* vinorine synthase (*Rs*VS) (PDB ID: 2BGH), a globular protein consisting of two nearly equal-sized domains connected by a crossover loop (amino acids 201–213) consisting of 14 β-strands (β1–β14) and 13 α-helixes (α1–α13) (**Figure 2A**). The HXXXD motif is located at the active center between the two domains, while the DFGWG motif is located at the intersection between β11 and β13, far from the active site. Both domains play an important role in maintaining the catalytic function and binding to the donors and substrates (Ma et al., 2005). The first characterized crystal structure of N-acyltransferase (refers to the acyltransferases using nitrogenous metabolites as substrates) in the BAHD family was of *Hordeum vulgare* agmatine coumaroyltransferase (*Hv*ACT) (PDB ID: 7CYS). The structure shares some commonality with *Rs*VS, that is, they both consist of two domains connected by a long and large crossover loop, but the *Hv*ACT contains 18 β-strands (β1–β18) and 13 α-helixes (α1–α13) (Yamane et al., 2020) (**Figure 2B**). The crystal structures of BAHD members have been published one after another, and the number of described structures is currently 26 (Garvey et al., 2008; Garvey et al., 2009; Lallemand et al., 2012; Manjasetty et al., 2012; Walker et al., 2013; Eudes et al., 2016b; Levsh et al., 2016; Chiang et al., 2018). The clarity of the crystal structures of BAHD acyltransferases contributes to the understanding of the conserved domains shared by the BAHD family and provides a basis for the exploration of catalytic mechanisms.

### 2.2 Catalytic mechanisms

The acylation mediated by BAHD acyltransferases involves CoA thioesters as the acyl donor, including acetyl-CoA, malonyl-CoA, succinyl-CoA, benzoyl-CoA, cinnamoyl-CoA, feruloyl-CoA, caffeoyl-CoA, sinapoyl-CoA, and coumaroyl-CoA. Moreover, these BAHD acyltransferases typically utilize alcohols as acceptors to generate the corresponding esters, including flavonoids, anthocyanins, and terpenoids, or use amines as acceptors to produce amide compounds, such as polyamines and alkaloids. Interestingly, the BAHD members differ greatly in their use of donors and acceptors. For example, alcohol acyltransferases are ubiquitous in plants, which accept diverse substrates for acylation, thus producing a variety of volatile lipids, including aromatic acid esters, short-chain fatty acid esters, and monoterpene esters (Hampel et al., 2009). In contrast, the acyltransferases involved in paclitaxel biosynthesis are found only in *Taxus* species, which form an independent phylogenetic branch and use specific substrates to produce taxanes (Kusano et al., 2019). Acylation enriches the structural diversity of the metabolites and improves their stability, lipid solubility, and bioavailability, which promotes their multiple functions in plant growth, as well as their potential applications in human healthcare (Liu et al., 2016a).

The research on crystal structures has improved the understanding of the acylation mechanism, and homology modeling and molecular docking have been widely used to study the acylation process and predict the potential functions of the products. According to the structural characteristics of *Rs*VS, Ma et al. (2005) proposed a catalytic mechanism in which the reaction is triggered by the histidine (His) residue of the HXXXD motif, which deprotonates the -NH_2_ and -OH on the substrates, allowing a nucleophilic attack on the carbonyl carbon of the CoA thioester. Next, a tetrahedral intermediate is formed between the thioesterase and the corresponding substrate, which re-protonates to produce the acylated ester or amide. As the most critical amino acid residue in acylation reaction, the His residue is absolutely conserved in all family member sequences, which was confirmed by site-directed mutagenesis of BAHD acyltransferases (Unno et al., 2007; Navarro-Retamal et al., 2016). Moreover, the aspartate (Asp) residue of the HXXXD motif seems to play a structural role, since it points away from the active site. Site-directed mutagenesis of converting Asp residue to amino acids with poor nucleophilic ability in the HXXXD motif of *Taxus cuspidata* 10-deacetylbaccatin III-10β-O-acetyltransferase (*Tc*DBAT) led to over 90% loss of enzyme activity (Wang et al., 2021a). A similar case was also reported by Morales-Quintana et al. (2013) in *Vasconcellea pubescens* alcohol acyltransferase (*Vp*AAT1), while *in vitro* site-directed mutagenesis of H166 and D170 in the HXXXD motif of *Vp*AAT1 induced a loss of activity, confirming the role of these amino acid residues that were directly involved in catalysis. An early mutagenesis study provided evidence that the DFGWG motif is necessary for catalysis and appears to have a structural function, while later studies used similar techniques suggested that the contribution of DFGWG motif was primarily binding to substrates rather than affecting catalytic efficiency (Morales-Quintana et al., 2015).

## 3 Phylogenetic reconstruction

**Figure 3:**
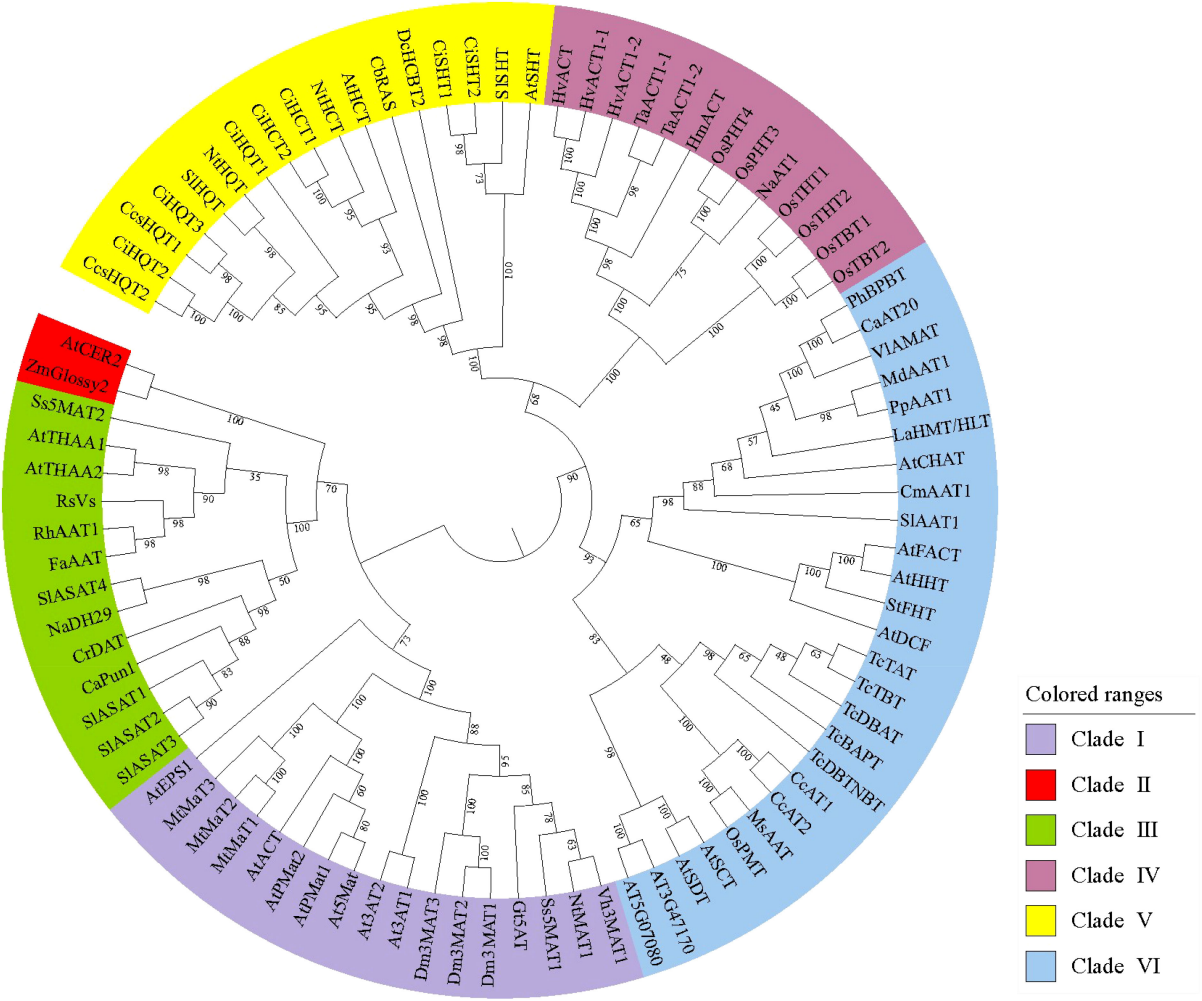
Maximum-likelihood phylogenetic tree of plant acyltransferases. These sequences were aligned using ClustalW as implemented in MEGA 11. Evolutionary analyses were conducted in MEGA 11 with 1000 bootstrap replicates, and the evolutionary tree was visualized by iTOL. Details of these sequences are list in **Supplemental Table S1**.

Phylogenetic analyses of the BAHD family have shown different results, possibly due to the different software and criteria used. D’Auria (2006) was the first to divide the 46 family members into five clades according to the type of substrate used or the conditions under which the genes and enzymes are active. Tuominen et al. (2011) reconstructed phylogenetic relationships using 69 known functional BAHD acyltransferases and other not functionally characterized putative members from Poplar, Arabidopsis, Oryza, Medicago, and Vitis, and obtained results wherein, although consistent with those reported by D’Auria, the family members were divided into eight main clades. Wang et al. (2022) used the amino acid sequences of 129 acyltransferases characterized between 1997 and 2020 to construct a BAHD phylogenetic tree using the maximum-likelihood method and proposed that the type of acceptors should be the main basis for the phylogeny of BAHDs. Moreover, Moghe et al. (2022) listed hydroxycinnamoyl-CoA:shikimate hydroxycinnamoyl transferase (HST) and hydroxycinnamoyl-CoA:quinate hydroxycinnamoyl transferase (HQT) as an independent clade based on the reports by D’Auria (2006). Another clade was added for algae acyltransferases and a loosely defined clade with coniferyl alcohol acetyltransferases as the main members, thus further describing the evolutionary relationships of these enzymes. Plant evolution is thought to have resulted in neofunctionalization by enzyme promiscuity and substrate permissiveness, which led to the synthesis of new metabolites and the establishment of related biosynthetic pathways (Fani and Fondi, 2009), while taxon-specific BAHD family expansion via gene duplication could be an evolutionary process contributing to metabolic diversity across plant taxa (Tuominen et al., 2011). This could explain the rapid expansion of metabolic pathways based on different substrates mediated by the BAHD family. In this review, an overview of the acylation reactions involving different family members is presented through different clades of the BAHD family, and representative amino acid sequences were selected for phylogenetic tree construction, which was used to reveal the functional evolution of BAHD family members and predict the function and activity of unidentified proteins (**Figure 3**).

### 3.1 Clade I – Synthesis of flavonoids/anthocyanins/phenolic glucosides

The members of Clade I are mostly involved in the acylation of flavonoids, anthocyanins, and phenolic glucosides, in particular, the acylation of anthocyanins. The proteins contain the YFGNC motif, which is a signature sequence of anthocyanin acyltransferases (Yu et al., 2009). The acylation of flavonoids generally occurs on the C6-OH of the glucosyl group, which possess region-specificity of acyl transfer (D’Auria, 2006). Based on the specificity of the donors, these mediated BAHDs are often classified as aromatic and aliphatic acyltransferases (Nakayama et al., 2003). Moreover, most flavonoid acyltransferases exhibit a wide range of acceptor availability that using glycosylated anthocyanins, isoflavons, flavanones, flavanols, and flavones as acceptors, and resulting in a huge diversity of potential products (Saigo et al., 2020). For example, *Arabidopsis thaliana* acyltransferase 1 (*At*3AT1) and acyltransferase 2 (*At*3AT2), which can utilize more than seven flavonoid 3/7-O-glucosides as substrates for acylation (Luo et al., 2007). Structural analysis of *Gentiana triflora* anthocyanin 5,3-aromatic acyltransferase (Gt5,3’AT) (PDB ID: 7DEV) reveals the residues responsible for acyl acceptor specificity and indicates that the selectivity for acyl transfer is determined by the C-terminal lobe of the protein (Murayama et al., 2021).

### 3.2 Clade II – Elongation of epicuticular waxes

Clade II mainly contains *Arabidopsis thaliana* Eceriferum (*At*CER2), *Zea mays* Glossy2 (*Zm*Glossy2), and *Zm*Glossy2-like, which are involved in the elongation of epicuticular waxes for preventing tissue moisture loss and resisting pathogen attack (Yu et al., 2009). However, Clade II proteins could not meet the original criteria of containing both HXXXD and DFGWG motif, and their functional activities need further investigation, so it is still unclear whether they belong to the BAHD family. Glossy2-like and Glossy2 functionally complement the *At*CER2 mutation, indicating a conserved function, while differences in the utilization of longer alkyl-chain acyl lipids indicate functional differentiation of these two maize enzymes (Alexander et al., 2020). In addition, based on mutation studies, a series of CER2-like proteins essential for epidermal wax production were characterized in *Arabidopsis thaliana* and *Zea mays*. Interestingly, CER2-like proteins possess sequence similarity with CER2, and make a major contribution to the chain-length specificity of fatty acid elongation (Haslam et al., 2017), but they seem to not rely on acyl transfer activity for their biological function (Haslam and Kunst, 2020). Further studies are expected to explore ligand binding and thiolipase activity, which will establish or rule out their most plausible functions for the protein family.

### 3.3 Clade III – A pluralistic clade

Clade III contains a series of alcohol acyltransferases involved in the biosynthesis of volatile lipids in flowers and mature fruits. Most of them use acetyl-CoA as the donor and accept different kinds of alcohol compounds as substrates, such as geraniol and *n*-octanol (Shalit et al., 2003). Interestingly, the alcohol acyltransferases often use various substrates and produce a wide range of products and, as a result, substrate promiscuity is considered a hallmark for this subset of BAHD members (Moghe et al., 2022). Moreover, this clade also contains proteins involved in the modification of alkaloids, such as vindoline, dimethylmorphine, and capsaicin (De Luca et al., 1985; Grothe et al., 2001; Milde et al., 2022). The alkaloids are a class of nitrogenous compounds, most of which possess a complex ring structure containing nitrogen and show significant biological activity (Mondal et al., 2019). The acyl transfer of the alkaloid-associated BAHD members depends on modification of the hydroxyl or amine moieties within the alkaloids. Recently, a series of acylsugar acyltransferases (ASATs) were classified into clade III. The ASATs are produced in type I/IV trichome tip cells and use acetyl or C2–C12 short-chain acyl groups to produce O-acylsugars (Schilmiller et al., 2012; Moghe et al., 2017). In theory, ASATs can produce various compounds, but the ultimate distribution of acylsugars within a species depends on the preferences of ASATs for substrates, which effectively narrows the acylsugar structural space (Taguchi et al., 2005; Unno et al., 2007). In addition, Clade III also contains various enzymes involved in the modification of anthocyanin glycoside, terpene, and aliphatic amine, which have been characterized in different angiosperm species, but their functional association was less clear due to the small size of subclades in this branch.

### 3.4 Clade IV – Agmatine coumaroyltransferases and putrescine hydroxycinnamoyltransferases

Several members of Clade IV have been reported, such as agmatine coumaroyltransferase (ACT) in barley (*Hv*ACT) and wheat (*Triticum aestivum* ACT, *Ta*ACT) (Kage et al., 2017; Yamane et al., 2020; Yamane et al., 2021). These enzymes contain the conserved HXXXD motif, but the DFGWG motif, which is shared by all members of the family, is slightly altered with glycine instead of tryptophan (Burhenne et al., 2003). The putrescine hydroxycinnamoyl- transferases (PHTs) have also been described in tobacco (*Nicotiana attenuata* acyltransferase, *Na*AT1), rice (*Oryza sativa* PHT 1-4, *Os*PHT1-4), and tomato (*Solanum lycopersicum* ACT, *Sl*ACT1) (Onkokesung et al., 2012; Campos et al., 2014; Fang et al., 2022). Both ACT and PHT enzymes can use a variety of amines as acceptors, including agmatine, putrescine, and spermidine, as well as donors such as 4-coumaryl-CoA, caffeoyl-CoA, feruloyl-CoA, and cinnamoyl-CoA, to generate corresponding amide compounds (Kruse et al., 2022). However, several N-acyltransferases have relatively high donor specificity. For example, *Os*TBT2 and *Os*PHT3 only use benzoyl-CoA and *p*-coumaryl-CoA as the donors, respectively; while *Hv*ACT exhibits donor preferences to *p*-coumaroyl-CoA (Burhenne et al., 2003).

### 3.5 Clade V – Hydroxycinnamoyl-CoA:shikimate acid hydroxycinnamoyl transferase and hydroxycinnamoyl-CoA:quinate acid hydroxycinnamoyl transferase

Clade V mainly contains hydroxycinnamoyl-CoA:shikimate acid hydroxycinnamoyl transferase (HST) and hydroxycinnamoyl-CoA:quinate acid hydroxycinnamoyl transferase (HQT). HST and HQT have hydroxycinnamoyl transferase (HCT) activity and use phenolic-CoA as donors, such as hydroxycinnamoyl-CoA, *p*-coumaryl-CoA, and caffeyl-CoA, to catalyze the acylation reaction with shikimic acid and quinic acid as substrates (Moglia et al., 2016). HST and HQT are involved in the phenylpropane pathway in plants, and their products are important intermediates in lignin synthesis, mediating plant growth and development (Kriegshauser et al., 2021). *In vitro* experiments have reported differences in the substrate selection of these enzymes, such as *Solanum lycopersicum* HQT (*Sl*HQT) and *Cynara cardunculus* HQT (CcsHQT) (Comino et al., 2007; Moglia et al., 2014), which only accept shikimic acid and/or quinic acid, while others do not strictly distinguish between these two substrates. Based on the crystal structure of HCT and mutagenesis experiments, this substrate specificity could be explained by the so-called “arginine handle” and the surrounding amino acids (Chiang et al., 2018). Interestingly, some enzymes, such as *Coleus blumei* rosmarinic acid synthase (*Cb*RAS) and *Coleus blumei* HST (*Cb*HST), can accept other nonrelated substrates, including 3-hydroxyanthranilate, 5-hydroxyanthranilate, 3-hydroxybenzoate, 2,3-dihydroxybenzoate, 3-aminobenzoate, gentisate, catechol, and protocatechuate (Sander and Petersen, 2011; Eudes et al., 2016a). Certainly, the reverse reactions catalyzed by these members also contribute to produce hydroxycinnamoyl-CoA and the former acceptor substrate or split hydroxycinnamoyl-CoA into free hydroxycinnamic acid and CoA (Berger et al., 2006).

### 3.6 Clade VI – A multicomponent clade

Members in Clade VI show diverse activities. They utilize substrates ranging from terpenoids to medium-chain alcohols, in association with major phylogenetic branches within this clade. Several enzymes are associated with the biosynthesis of volatile esters, including *Petunia hybrida* benzoyl-CoA:8-debenzoylpaeoniflorin 8-O-benzoyltransferase (*Ph*BPBT), *Arabidopsis thaliana* acetyl-CoA:(Z)-3-hexen-1-ol acetyltransferase (*At*CHAT), and *Solanum lycopersicum* alcohol acyltransferase 1 (*Sl*AAT1) (Okada et al., 2005; Molina and Kosma, 2015; Liu et al., 2016b). These enzymes are characterized by the ability to acylate aliphatic and/or aromatic alcohols using aliphatic and/or aromatic CoA-activated donors. Similar to the alcohol acyltransferases in Clade III, these enzymes show a wide substrate diversity, but aliphatic and aromatic acyl acceptors are not the main basis for their clade division. Specialized taxane acyltransferase-mediated acylation reactions are thought to be important steps in modifying the core backbone of paclitaxel, an important anticancer drug. To date, five key acyltransferases have been characterized as being involved in the paclitaxel pathway (Wang et al., 2021a), and these enzymes are clustered into an individual group within Clade VI. With the publication of the whole genome of *Taxus chinensis* var. *mairei*, as many as 53 candidate genes encoding taxane acyltransferases have been reported, which potentially enlarged the size of branch for taxane acyltransferases. It would be worthwhile to investigate whether these candidates function in paclitaxel biosynthesis in the future (Xiong et al., 2021). Members of Clade VI also include lipid-related acyltransferases, such as *Marchantia emarginata* ω-hydroxyacid/fatty alcohol hydroxycinnamoyltransferase (*Me*HFT) and *Marchantia polymorpha* feruloyltransferase (*Mp*FHT), which are involved in the biosynthesis of cutin monomers (Wang et al., 2017). These enzymes use aroyl-CoA as donors, including feruloyl-CoA, *p*-coumaroyl-CoA, caffeoyl-CoA, and sinapoyl-CoA, and accept fatty acids or fatty alcohols as substrates to produce alkyl hydroxycinnamates. Functionally, they are similar to those of Clade II, but orthologs of the characterized members of Clade II are found only in angiosperms. Moreover, the clade also includes the first anthraniloyl-CoA:methanol acyltransferase (AMAT) found in the BAHD family with O-aminobenzoylkylase A as the acceptor for synthesis of aromatic esters, as well as the *Lupinus albus* tigloyl-CoA: (–)- 13α-hydroxymultiflorine/(+)- 13α-hydroxylupanine O-tigloyltransferase (*La*HMT/HLT), which is related to the biosynthesis of quinolicidine alkaloids (Goulet et al., 2015; Huang et al., 2022).

### 3.7 Other members

A few acyltransferases show a specific distribution in different clades due to functional diversity and complex evolutionary dynamics of the BAHD family. For example, *Salvia splendens* anthocyanin 5-O-glucoside-4’’’-O-malonyltransferase 2 (*Ss*5MaT2), although associated with the modification of anthocyanins, was classified in Clade III because it does not contain the conserved motifs that are common in Clade I (Suzuki et al., 2004b). This also reflects different routes for anthocyanin acyltransferase activity. Due to the diversity of available substrates, members with HCT activity are found in different clades, except for Clade II, and differences are also observed in the number of hydroxycinnamoyl residues carried by the products (Roumani et al., 2021). The different groups that are modified in the alkaloids, as well as the wide diversity in the sources of precursors for nitrogenous heterocyclic compounds, have resulted in a scattered distribution of alkaloid acyltransferases throughout the BAHD family. Moreover, Moghe et al. (2022) reported an algal acyltransferase clade based on HQT activity and an undefined clade consisting of coniferyl alcohol acetyltransferases. Wang et al. (2022) reported a class of lipid-related acyltransferases that use long-chain fatty acids as donors and accepting glycerol derivatives as acceptors to form glycerolipids. Since their functions have not been fully determined using *in vitro* experiments, they were not classified in **Figure 3**.

## 4 Potential applications of BAHD acyltransferases

As we catalog below, BAHD members mediate diverse ecological interactions in plants to ensure their normal growth and development. For example, the BAHD family of proteins is involved in the following: improving plant pollination by forming brilliant colors and scented volatiles; supporting plant morphology by mediating lignin synthesis; protecting plant reproduction by mediating pollen wall formation; and resisting different biotic and abiotic stresses by forming a variety of secondary metabolites in different tissues. Given the enzyme promiscuity of the BAHD family and the rapid evolutionary neofunctionalization, understanding the BAHD family will enhance our knowledge of plant ecology. Moreover, these extensive acylated modifications may have applications in, for example, economic development and human healthcare. Here, we focus on the application prospects of the BAHD family in the development of human activities and summarize the functions of the BAHD family mediating the diverse plant traits.

### 4.1 Improving the quality of economic plants

Volatile esters play an important role in the formation of aromas in the plant leaf, flower, and fruit. The BAHD members involved in the synthesis of volatile esters are mainly from Clades III and VI, which possess the potential to enhance the quality of economic plants. For example, *Malus domestica* alcohol acyltransferase 2 (*Md*AAT2) is a key enzyme in the last step of apple volatile ester biosynthesis, which is a key factor in guaranteeing fruit quality (Li et al., 2006). Since alcohol acyltransferases mediate the synthesis of specific esters in different plants, which could be an important reason that causes different fruits to exude unique smells. For example, short-chain esters derived from fatty acids contribute to the characteristic flavor of pear, while its alcohol acyltransferase, *Pa*AAT1, is thought to be closely associated with the production of C6 esters (Zhou et al., 2021). The odor in peaches is mainly determined by γ-decalactone, whose synthesis is mainly catalyzed by *Prunus persica* alcohol acyltransferase (*Pp*AAT) (Song et al., 2021). The alcohol acyltransferases also demonstrate broad substrate diversity for driving the delicate development of characteristic odors. For example, overexpression of the gene encoding *Fragaria vesca* alcohol acyltransferase (*Fv*AAT) increases the proportion of different esters to volatiles, including octyl acetate, ethyl caproate, octyl hexanoate, and ethyl caprylate, which indicates that *Fv*AAT can influence the volatile ester composition of strawberry fruit (Dong et al., 2018). The *Vitis vinifera* alcohol acyltransferase (*Vv*AAT)-mediated ester components in grapes are important markers to distinguish different cultivars (Ji et al., 2022).

The formation of leaf and floral aromas has also received attention. The composition of *Cymbopogon winterianus* leaf oil mainly consists of acyclic monoterpenols (geraniol and citronellol) and their esters (geranyl acetate and citronellyl acetate), and the synthesis of these compounds is associated with citronellol alcohol acyltransferase (CAAT) (Kumar, 2020). The function of O*smanthus fragrans* alcohol acyltransferase1 (*Of*AAT1) was predicted to be similar to that of *Petunia hybrida* coniferyl alcohol acyltransferase (*Ph*CFAT), which can catalyze coniferol to produce coniferol diester that is further degraded to isoeugenol. However, it has also been documented that *Of*AAT1 might be involved in the synthesis of fatty acid esters. The mechanism is the same as that of *Glycine max* cholineacetyltransferase (*Gm*CHAT) and *Solanum tuberosum* cholineacetyltransferase (*St*CHAT) (Liu et al., 2016b). Benzylalcohol *O*-acetyltransferase uses benzyl alcohol and acetyl-CoA to produce benzyl acetate, a floral volatile found in a few plants, including *Clarkia pulchella*, *Prunus mume*, and *Chimonanthus praecox*. Interestingly, BEAT is also capable of accepting other alcohols as substrates, but the highest catalytic efficiency is achieved when benzyl alcohol is the substrate (Dudareva et al., 1998). These studies provide important clues to the potential phylogeny and functional diversity of alcohol acyltransferases.

Anthocyanins are natural water-soluble pigments in plants, including delphinidin, cyanidin, and pelargonidin, which are catalyzed by glycosyltransferases to form a variety of anthocyanin glycosides with different colors stored in vacuoles; while the latter are further modified by methyltransferases and acyltransferases, and through molecular superposition and interaction effects, ultimately produce different colors in organs and tissues of different plants (Zhao and Tao, 2015). Florio et al. (2021) reported that the expression level of the *Solanum melongena* anthocyanin acyltransferase (*SmelAAT*) gene correlated with the accumulation of lycopene in the pericarp, which provides key evidence for the involvement of *Smel*AAT in anthocyanin modification. Anthocyanin acyltransferases also exhibit diverse preferences for the combination of donors and substrates. Among a host of characterized anthocyanin acyltransferases, the *Vitis vinifera* anthocyanin acyltransferases (*Vv*3AT), reported by Rinaldo et al. (2015), showed a preference for anthocyanin monoglycoside molecules, allowing the use of aromatic and aliphatic acyl-CoA thioesters as substrates, which was different from the previously characterized natural BAHD acyltransferase. Suzuki et al. (2004a) reported that *Dendranthema morifolium* malonyl-CoA:anthocyanidin 3-O-glucoside-6″-O-malonyltransferase (*Dm*3MaT2) could catalyze the malonylation of anthocyanidin 3-O-glucoside to produce anthocyanidin 3-O-6″-O-malonylglucoside and subsequently catalyze the latter to produce anthocyanidin 3-O-3″,6″-O-dimalonylglucoside. Extensive studies have shown that aromatic acylation of anthocyanins through intramolecular condensation leads to a more stable molecular structure and coloring, while fatty acid acylation increases the water solubility of the compounds, protects the glycosides from enzymatic hydrolysis, stabilizes the structure, and contributes to the accumulation of the compounds in the vesicles (Bloor and Abrahams, 2002; Luo et al., 2007; Zhang et al., 2014). Therefore, in-depth studies on the catalytic pattern of anthocyanin acyltransferases in plants will help to improve the external and internal qualities and promote bioengineering to produce anthocyanins.

### 4.2 Enhancing the efficacy of medicinal plants

Acylation plays an important role in the structural modification and pharmacological activity of plant secondary metabolites and is crucial for obtaining structural diversity and active medicinal lead compounds. A series of BAHD members participate in the synthesis of alkaloids and terpenoids, which are very important components of herbal medicines, with a broad spectrum of antitumor, antiviral, antibacterial, antimalarial, and analgesic biological and pharmacological activities (Yu et al., 2009). Vinblastine is a clinically important antitumor chemotherapeutic agent. Vincenzo et al. (1985) first reported that acetyl coenzyme A: deacetylvincristine O-acetyltransferase is involved in the formation of vindoline, a precursor of vincristine, which initiated the research on the synthesis and modification of alkaloids mediated by BAHD acyltransferases (Grothe et al., 2001). *Rs*VS is a key enzyme involved in the biosynthesis of the antiarrhythmic drug ajmaline that can reversibly catalyze the synthesis of the sarpagan alkaloid 16-epi-vellosimine with acetyl-CoA to obtain the ajmalan-like alkaloid vinoline. As the first reported crystal structure of the BAHD family, *Rs*VS confirms the shared key properties of the BAHD family. Grothe et al. (2001) found that *Papaver somniferum* salutaridinol-7-O-acetyltransferase (*Ps*SALAT) catalyzes the phenanthrene alkaloid salutaridinol to produce salutaradinol-7-O-acetate, which is an immediate precursor of morphine. Capsaicin has antioxidant and anticancer effects, and the final step of its biosynthetic pathway involves the N-acylation of vanillin with a variable-length fatty acyl-CoA donor. However, the capsaicin synthase Pun1 is highly insoluble, and its role in capsaicin accumulation is based exclusively on genetic studies and no biochemical activity has been reported. The pharmacologically important anticancer drug paclitaxel is a complex diterpene alkaloid whose biosynthesis involves five acyl modifications. Among them, taxadiene-5α-ol-O-acetyl transferase (TAT) catalyzes the first acylation reaction of the paclitaxel pathway, converting taxadiene-5-α-ol to taxadiene-5-α-acetate, which is considered a slow step of the downstream hydroxylation reaction, while 10-deacetylbaccatin III-10-O-acetyl transferase (DABT) is the key rate-limiting enzyme of the paclitaxel pathway, acetylating the C-10 hydroxyl group of 10-deacetylbaccatin III (10-DAB) to produce baccatin III, an important intermediate of taxol (Wang et al., 2021a).

Due to the low content of natural medicinal active ingredients in plants, the overexpression of the corresponding genes by means of genetic transformation techniques or the reorganization of synthetic pathways using different plants or microorganisms is considered the most promising method to enhance the yield and quality of the products. However, the existing genetic transformation systems, such as the suspension cell line of Taxus and the hairy root pathway mediated by *Agrobacterium perfringens* in periwinkle, are difficult to apply at industrial scales due to factors such as the slow cell growth and unstable production capacity. Moreover, transient acylation and deacylation occur in the synthetic pathways of the above-mentioned medicinal components for flux regulation of organelle targeting, but the promiscuity and preferences of the corresponding acyltransferases have not been fully investigated (McElroy and Jennewein, 2018). Furthermore, most acyltransferases for terpenoids or alkaloids that are heterologously expressed in different engineered strains, such as *Escherichia coli* and *Saccharomyces cerevisiae*, still have drawbacks such as low expression levels, incompatible solubility, and poor stability (Wang et al., 2022). Together with the long synthetic pathways, these factors make it hard to obtain desirable products from heterologous synthesis, or the purification of products cannot meet the actual demand. There is still a long way to go before BAHD members can be efficiently utilized in synthetic biology and metabolic engineering.

### 4.3 Improving plant biomass for use in biofuel

The manipulation of cell wall polymers can produce plants that are useful for biofuel production. Lignin is a principal structural component of cell walls and is formed from the polymerization of single lignin alcohols (coumaryl alcohol, coniferyl alcohol, and sinapyl alcohol) to produce different lignin units. Coumaryl alcohol produces 4-hydroxyphenyl (H) units, and coniferyl alcohol and sinapyl alcohol produce guaiacyl (G) and syringyl (S) units, respectively (Vanholme et al., 2019). Lignin levels affect plant quality, and excessive lignin levels can cause reduced digestibility. HCT is one of the key enzymes affecting the biosynthesis of lignin G/S units, and research on HCT has focused on its association with lignin (Kriegshauser et al., 2021). A low level of HCT leads to a decrease in lignin content and results in a significant increase in biomass saccharification efficiency (Serrani-Yarce et al., 2021). However, it also leads to drastic changes in the plant phenotype. Based on the potential functions of HSTs in the pathways of phenylpropanoid metabolism and hormone response, Dong et al. (2022) suggested that HCTs may influence plant lignin composition and development by altering hormone content. Notably, some BAHD members involve the modification of monolignol monomers, which are then incorporated into lignin polymers as acyl conjugates. For example, *Angelica sinensis* feruloyl-CoA monolignol transferase *(As*FMT), which specifically accepts Feruloyl-CoA as the acyl donor but can accept all three monolignol monomers as substrates, shows an increase in saccharification yield in poplar strains with increased levels of feruloylated conjugates mediated by BAHD (Wilkerson et al., 2014). Liu et al. (2022b) reported the crystal structure of *As*FMT, and revealed the key action sites concerning its mediated acylation reaction, which provides insights into the formation of monolignol ferulate conjugates. These data may help in the design of strategies to optimize the lignin composition and amount in biorefining.

BAHD family also plays important roles in the addition of phenolic acids, such as ferulic acid (FA) and *p*-coumaric acid (pCA) to form ester-linked moieties on the xylan backbone of glucuronoarabinoxylan (GAX) (Chandrakanth et al., 2023). In the complex structure of plant cell walls, cellulose is protected by a network of xylan chains that cross-link via FA or lignin. The role of pCA on GAX is less obvious than that of FA, because the oxidative coupling of pCA is much weaker than that of FA. One possibility is that pCA-GAX participates in radical transfer and thus catalyzes the oxidative coupling of neighboring FA on GAX. The degree of ferulic acidification in the cell wall is related to biomass digestibility, as high FA increases the resistance of biomass conversion to ethanol (de Oliveira et al., 2015). *Oryza sativa* acyltransferase 10 (*Os*AT10) was the first reported putative *p*-coumaroyl CoA arabinofuranose transferase (PAT), since overexpression of *OsAT10* induced a 5-fold increase in pCA levels in young green tissues with a concomitant 50% decrease in FA linked to GAX (Bartley et al., 2013). Interestingly, overexpression of *OsAT10* in *Sorghum bicolor* and *Panicum virgatum* and of Sugarcane *AT10* (*ScAT10*) in maize also significantly improve the biomass saccharification efficiency with increased *p*-CA in transgenic strains, but not universally accompanied by a decrease in FA-GAX (Li et al., 2018; Fanelli et al., 2021; Tian et al., 2021). Silencing of the putative FAT-encoding *Setaria viridis* acyltransferase 01 (*Sv*BAHD01/*Sv*AT9) gene decreased arabinoxylan feruloylation by 60% and increased biomass saccharification efficiency by 40–60% in the stems without changing the amount of lignin (de Souza et al., 2018). Similarly, suppression of the ortholog in Sugarcane (*ScBAHD01/ScAT9*) improved the digestibility of sugarcane straw by approximately 20% compared to non-transformed plants (de Souza et al., 2019). These results further indicate that the exploitation of BAHD acyltransferases for plant modification to improve biomass digestibility and to generate optimal biofuel plants is an important challenge for the sustainable production of advanced biofuels.

### 4.4 Promoting the stress resistance of land plants

Plant BAHD acyltransferases are involved in the synthesis and modification of a wide range of primary and secondary metabolites, thus enhancing the resistance of plants to different biotic and abiotic stresses in different dimensions, which is essential to guarantee their survival and yield. On the one hand, the BAHD proteins could mediate the synthesis of many chemical substances to increase tolerance to various environmental stresses. For example, the resistance of plants to UV radiation is achieved by the BAHD members that mediate the synthesis of anthocyanins, and the resistance benefits from the formation of the so-called bridge-piled structure, leading to an intramolecular co-pigmentation-like effect (de Oliveira et al., 2015; Tohge et al., 2016). Overexpression of the gene that encodes *Taraxacum mongolicum* HQT (*Ta*HQT) increases the chlorogenic acid (CGA) content, which is used to enhance disease resistance and salt tolerance, in the transgenic plants (Liu et al., 2018). On the other hand, The BAHD acyltransferases could also activate multiple metabolic pathways simultaneously to improve plant stress resistance. For example, *Oryza sativa* acyltransferase (*Os*At10) can promote the growth rate and viability of cells, and it improves cold tolerance in *Arabidopsis*, cotton, and poplar by increasing the antioxidative enzyme activity and polyamine level. This provides a valuable genetic resource for breeding cultivars with high yield and high resistance (Tang and Thompson, 2022). Moreover, the *Camellia sinensis HCT* (*CsHCT*) gene is expressed under low temperature, drought, and high salinity environments and is involved in the response to abiotic stresses (Sun et al., 2018). The formation of epidermal waxes mediated by CER2-like proteins may mediate water rejection, particle adhesion, light reflection, and herbivore resistance of plant cells. There could also be crosstalk among different environmental stresses, that is, enhancing resistance to a single environmental stress increases resistance to other stresses at the same time (Haslam et al., 2015).

Pests and diseases are important limiting factors affecting crop yield and their ecological roles. Traditional chemical control enhances pest and disease resistance but affects the ecological balance and human health. Among the characterized BAHD acyltransferases, some clade members specifically mediate plant responses to pests and diseases and are the focus of research in the field of biological control. As an important chemical defense compound in plants, hydroxycinnamic acid amide exhibits a pivotal role in plant–pathogen interactions, and its antimicrobial activity and the mechanism involved in plant immune response have been fully elaborated (Liu et al., 2022a). Hydroxycinnamic acid amides are synthesized through the condensation of various biogenic amines with hydroxycinnamic acids via BAHD acyltransferases. ACT was first characterized in barley, catalyzing agmatine and hydroxycinnamoyl-CoA to produce hydroxycinnamoyl agmatine, the precursor of Hordatine, which is an antifungal compound that is abundant in yellowing barley seedlings (Burhenne et al., 2003; Ube et al., 2017). Homologous genes of ACT in Arabidopsis, Rice, and Wheat have been reported (Onkokesung et al., 2012; Peng et al., 2016; Kage et al., 2017; Yamane et al., 2021), and numerous studies have confirmed that ACT can mediate the synthesis of neurotoxins similar to those found in spider and wasp venom to promote immunity against pathogenic bacteria and herbivorous pests (Dobritzsch et al., 2016; Bai et al., 2022). Hydroxycinnamoyl-coenzyme A (CoA): malate hydroxycinnamoyl transferase (HMT) and hydroxycinnamoyl-CoA: L-DOPA hydroxycinnamoyl transferase (HDT) were successively characterized in red clover leaves, which mediated the synthesis of phaselic acid and clovamide, protecting the plant from biotic and abiotic stresses. Notably, they share a 72% degree of identity, but differ substantially in substrate specificity, which provides valuable and new directions for rational design of BAHD enzymes with specific and desirable activities (Sullivan and Knollenberg, 2021). O-Acyl sugars produced by glandular trichomes are defense molecules against fungal pathogens and a chemical glue for trapping small insects. *Nicotiana tabacum* acyl-glycoacyltransferase 1 (*Nt*ASAT1) and acyl-glycoacyltransferase 2 (*Nt*ASAT2) are key enzymes involved in acylsugar assembly in tobacco and mediate a post-ingestive odor-tagging indirect defense mechanism, which provides new pathways and target genes for plant resistance against pathogens and insects (Chang et al., 2022). Moreover, members of Clade II, as well as lipid-related acyltransferases in Clade VI, such as *Arabidopsis thaliana* aliphatic suberin feruloyl transferase (*At*ASFT), are involved in the synthesis of plant cell membranes, keratin, and suberin. These substances provide a protective layer on the surface of plants to maintain water homeostasis and provide protection against pathogens. In summary, plant BAHD acyltransferases regulate the accumulation of different metabolites via acylation, thus protecting plants from external environmental changes, which provides new directions for plant ecology and genetic improvement.

## 5 Summary and prospects

BAHD acyltransferases play an important role in plant growth and development, stress responses, and synthesis and modification of secondary metabolites. In recent years, with the completion and improvement of plant genome sequencing and assembly, and the development of molecular biology techniques, such as molecular docking, homology modeling, RNA interference, targeted mutagenesis, and molecular dynamics simulation, an increasing number of BAHD family members have been discovered and characterized, laying a solid theoretical foundation for further elucidation of the biological functions of the plant BAHD family. Given the extensive potential applications of plant BAHD acyltransferases in human activities, there is still ample room for further exploration of BAHD acyltransferases. Currently, despite the increasing numbers of annotation of BAHD members, studies on their biochemical functions are limited. From a phylogenetic perspective, the role of a series of subfamily members in the evolution of plant geographic lineages remains to be further explored. Moreover, the biochemical properties of BAHD acyltransferases should be the focus of attention. The selection and preferences of different members for substrates and donors, as well as the construction of suitable *in vivo* and *in vitro* reaction systems, could contribute to the rapid development of BAHD acyltransferases in synthetic biology and metabolic engineering and promote their potential application.

## Author contributions

DX contributed to conception and design of the study and wrote the manuscript. WZ and ZW organized the database. TW and YX read, and approved the submitted version. All authors contributed to manuscript revision, read, and approved the submitted version.
