# Supplementary material for: Family characteristics, phylogenetic reconstruction, and potential applications of the plant BAHD acyltransferase family
Source: Front Plant Sci. 2023 Oct 5;14:1218914. doi: 10.3389/fpls.2023.1218914 (PMC10585174; doi:10.3389/fpls.2023.1218914)
Supplement: Supplementary file 1 [file Table_1.docx]

Supplemental Table 1 Biochemically characterized BAHD acyltransferases.

| Enzyme name | Accession number | Species | Acceptor | Donor | Reference |
| --- | --- | --- | --- | --- | --- |
| At3AT1 | NP_171890.1 | Arabidopsis thaliana | cyanidin 3-O-glucoside (<https://pubchem.ncbi.nlm.nih.gov/compound/cyanidin-3-glucoside>)  pelargonidin 3-O-glucoside (<https://pubchem.ncbi.nlm.nih.gov/compound/Kaempferol-7-o-D-glucopyranoside>)  malvidin 3-O-glucoside (<https://pubchem.ncbi.nlm.nih.gov/compound/Malvidin-3-Glucoside>)  kaempferol 7-O-glucoside (<https://pubchem.ncbi.nlm.nih.gov/compound/Kaempferol-7-o-D-glucopyranoside>)  quercetin 3-O-glucoside (<https://pubchem.ncbi.nlm.nih.gov/compound/quercetin-3_-O-glucoside>) | Malonyl CoA | (Luo et al., 2007) |
| At3AT2 | NP_171849.3 | Arabidopsis thaliana | cyanidin 3-O-glucoside (<https://pubchem.ncbi.nlm.nih.gov/compound/cyanidin-3-glucoside>)  pelargonidin 3-O-glucoside (<https://pubchem.ncbi.nlm.nih.gov/compound/Kaempferol-7-o-D-glucopyranoside>)  malvidin 3-O-glucoside (<https://pubchem.ncbi.nlm.nih.gov/compound/Malvidin-3-Glucoside>)  kaempferol 7-O-glucoside (<https://pubchem.ncbi.nlm.nih.gov/compound/Kaempferol-7-o-D-glucopyranoside>)  quercetin 3-O-glucoside (<https://pubchem.ncbi.nlm.nih.gov/compound/quercetin-3_-O-glucoside>) | Malonyl CoA | (Luo et al., 2007) |
| AT3G47170 | NP_190301.2 | Arabidopsis thaliana | putrescine (<https://pubchem.ncbi.nlm.nih.gov/substance/24278650>) | Caffeoyl-CoA | (Wang et al., 2020) |
| AT5G07080 | OAO95042.1 | Arabidopsis thaliana | putrescine (<https://pubchem.ncbi.nlm.nih.gov/substance/24278650>)  spermidine (<https://pubchem.ncbi.nlm.nih.gov/compound/spermidine>) | Feruloyl-CoA | (Wang et al., 2020) |
| At5Mat | NP_189600.1 | Arabidopsis thaliana | cyanidin 3,5-O-diglucoside (<https://pubchem.ncbi.nlm.nih.gov/compound/Cyanidin-3_5-diglucoside>)  malvin (<https://pubchem.ncbi.nlm.nih.gov/compound/Malvin>)  cyanin (<https://pubchem.ncbi.nlm.nih.gov/compound/Cyanin>) | Malonyl- CoA | (D’Auria et al., 2007) |
| AtACT | NP_200924.1 | Arabidopsis thaliana | agmatine (<https://pubchem.ncbi.nlm.nih.gov/compound/agmatine>)  putrescine (<https://pubchem.ncbi.nlm.nih.gov/substance/24278650>) | Feruloyl-CoA | (Muroi et al., 2009) |
| AtASFT | AAL34170.1 | Arabidopsis thaliana | 16-Hydroxypalmitic acid (<https://pubchem.ncbi.nlm.nih.gov/compound/16-hydroxypalmitate>)  15-Hydroxypentadecanoic acid (<https://pubchem.ncbi.nlm.nih.gov/compound/15-Hydroxypentadecanoic-acid>) | Feruloyl-CoA | (Molina et al., 2009) |
| AtCER2 | CAA63618.1 | Arabidopsis thaliana | C30 epicuticular waxes | unknow | (Negruk et al., 1996) |
| AtCHAT | AAN09797.1 | Arabidopsis thaliana | benzoyl alcohol (<https://pubchem.ncbi.nlm.nih.gov/compound/Benzyl-alcohol>)  1-Butanol (<https://pubchem.ncbi.nlm.nih.gov/compound/1-butanol>)  1-Octanol (<https://pubchem.ncbi.nlm.nih.gov/compound/1-octanol>)  2-Phenylethanol (<https://pubchem.ncbi.nlm.nih.gov/compound/2-phenylethanol>)  1-Hexanol (<https://pubchem.ncbi.nlm.nih.gov/compound/1-Hexanol>)  1-Heptanol (<https://pubchem.ncbi.nlm.nih.gov/compound/1-heptanol>)  1-Decanol (<https://pubchem.ncbi.nlm.nih.gov/compound/1-decanol>)  geraniol (<https://pubchem.ncbi.nlm.nih.gov/compound/geraniol>)  (Z)-3-hexen-1-ol (<https://pubchem.ncbi.nlm.nih.gov/#query=3-Hexenol>)  cinnamyl alcohol (<https://pubchem.ncbi.nlm.nih.gov/compound/Cinnamyl-alcohol>)  (e)-2-hexen-1-ol (<https://pubchem.ncbi.nlm.nih.gov/compound/5318042>) | Acetyl-CoA | (D’Auria, 2006) |
| AtDCF | NP_201161.1 | Arabidopsis thaliana | 16-hydroxypalmitic acid (<https://pubchem.ncbi.nlm.nih.gov/compound/16-hydroxypalmitate>) | Feruloyl-CoA,  sinapoyl-CoA | (Rautengarten et al., 2012) |
| AtEPS1 | NP_201517.1 | Arabidopsis thaliana | Isochorismoyl-glutamate A  isochorismoyl-glutamate B | none | (Torrens-Spence et al., 2019) |
| AtFACT | NP_851111.1 | Arabidopsis thaliana | Fatty alcohol (<https://pubchem.ncbi.nlm.nih.gov/compound/2724258>) | Caffeoyl-CoA,  feruloyl-CoA,  coumaroyl-CoA | (Kosma et al., 2012) |
| AtHCT | NP_199704.1 | Arabidopsis thaliana | Shikimate (<https://pubchem.ncbi.nlm.nih.gov/compound/Shikimic-acid>) | Coumaroyl-CoA | (Hoffmann et al., 2005) |
| AtHHT | ACY78659.1 | Arabidopsis thaliana | 16-OH-palmitic acid (<https://pubchem.ncbi.nlm.nih.gov/compound/Palmitic-acid>) | Feruloyl-CoA,  coumaroyl-CoA | (Gou et al., 2009) |
| AtPMat1 | AAK96528 .1 | Arabidopsis thaliana | 2NAG (<https://pubchem.ncbi.nlm.nih.gov/compound/139043311>)  kaempferol 7-O-glucoside (<https://pubchem.ncbi.nlm.nih.gov/compound/Kaempferol-7-o-D-glucopyranoside>) | Malonyl-CoA | (Taguchi et al., 2010) |
| AtPMat2 | NP_189609 .1 | Arabidopsis thaliana | 4-Methylumbelliferone glucoside (<https://pubchem.ncbi.nlm.nih.gov/compound/4-Methylumbelliferyl-glucoside>)  phenyl glucoside (<https://pubchem.ncbi.nlm.nih.gov/compound/Prenyl-glucoside>) | Malonyl- CoA | (Taguchi et al., 2010) |
| AtSCT | NP_180087 .1 | Arabidopsis thaliana | Spermidine (<https://pubchem.ncbi.nlm.nih.gov/compound/spermidine>) | Coumaroyl-CoA | (Luo et al., 2009) |
| AtSDT | NP_179932.1 | Arabidopsis thaliana | Putrescine (<https://pubchem.ncbi.nlm.nih.gov/substance/24278650>),  Spermidine (<https://pubchem.ncbi.nlm.nih.gov/compound/spermidine>) | Coumaroyl-CoA | (Wang et al., 2020) |
| AtSHT | NP_179497.1 | Arabidopsis thaliana | Spermidine (<https://pubchem.ncbi.nlm.nih.gov/compound/spermidine>) | Feruloyl-CoA | (Grienenberger et al., 2009) |
| AtTHAA1 | AAO64765.1 | Arabidopsis thaliana | 16-keto-3β,7β,15-thaliantriol (T6) | Acetyl-CoA | (Huang et al., 2019) |
| AtTHAA2 | AAO63428.1 | Arabidopsis thaliana | Thalaniol (T1) (<https://pubchem.ncbi.nlm.nih.gov/compound/Thalianol>) | Acetyl-CoA | (Huang et al., 2019) |
| MsAAT | CAC09063.1 | Musa sapientum | Geraniol (<https://pubchem.ncbi.nlm.nih.gov/compound/geraniol>)  1-Octanol (<https://pubchem.ncbi.nlm.nih.gov/compound/1-octanol>) | Acetyl-CoA,  hexanoyl-CoA,  butanoyl-CoA | (Beekwilder et al., 2004) |
| CaAT20 | QLI57449.1 | Solanum lycopersicum | Geraniol (<https://pubchem.ncbi.nlm.nih.gov/compound/geraniol>) | Benzoyl-CoA,  acetyl-CoA | (Yan et al., 2020) |
| CbRAS | CAK55166.1 | Coleus blumei Benth | DHPLA (<https://pubchem.ncbi.nlm.nih.gov/substance/57391917>)  HPLA (<https://pubchem.ncbi.nlm.nih.gov/substance/6448>) | Coumaroyl-CoA,  caffeoyl-CoA | (Berger et al., 2006) |
| CcAT1 | AXB26761.1 | Crocosmia crocosmiiflora | Quercetin 3-O- sophoroside (<https://pubchem.ncbi.nlm.nih.gov/compound/Quercetin-3-sophoroside>)  myricetin-Rhammnose-Glucose (<https://pubchem.ncbi.nlm.nih.gov/compound/25230470>) | Caffeoyl-CoA,  coumaroyl-CoA,  feruloyl-CoA | (Irmisch et al., 2018) |
| CcAT2 | AXB26762.1 | Crocosmia crocosmiiflora | Quercetin 4-O- sophoroside (<https://pubchem.ncbi.nlm.nih.gov/compound/Quercetin-3-sophoroside>)  myricetin-Rhammnose-Glucose (<https://pubchem.ncbi.nlm.nih.gov/compound/25230470>) | Caffeoyl-CoA,  coumaroyl-CoA,  feruloyl-CoA | (Irmisch et al., 2018) |
| CcsHQT1 | CAM84302.2 | Cynara cardunculus | Quinate (<https://pubchem.ncbi.nlm.nih.gov/compound/Quinate>)  Shikimate (<https://pubchem.ncbi.nlm.nih.gov/compound/Shikimic-acid>)  CGA (<https://pubchem.ncbi.nlm.nih.gov/substance/57654010>) | Caffeoyl-CoA,  coumaroyl-CoA | (Sonnante et al., 2010) |
| CcsHQT2 | ACJ23164.1 | Cynara cardunculus | Quinate (<https://pubchem.ncbi.nlm.nih.gov/compound/Quinate>)  Shikimate (<https://pubchem.ncbi.nlm.nih.gov/compound/Shikimic-acid>)  CGA (<https://pubchem.ncbi.nlm.nih.gov/substance/57654010>) | Caffeoyl-CoA,  coumaroyl-CoA | (Sonnante et al., 2010) |
| CiHCT1 | ANN12609.1 | Cichorium intybus | Shikimate (<https://pubchem.ncbi.nlm.nih.gov/compound/Shikimic-acid>)  quinate (<https://pubchem.ncbi.nlm.nih.gov/compound/Quinate>) | Caffeoyl-CoA,  coumaroyl-CoA | (Legrand et al., 2016) |
| CiHCT2 | ANN12608.1 | Cichorium intybus | Shikimate (<https://pubchem.ncbi.nlm.nih.gov/compound/Shikimic-acid>)  quinate (<https://pubchem.ncbi.nlm.nih.gov/compound/Quinate>) | Caffeoyl-CoA,  coumaroyl-CoA | (Legrand et al., 2016) |
| CiHQT1 | ANN12610.1 | Cichorium intybus | Quinate (<https://pubchem.ncbi.nlm.nih.gov/compound/Quinate>) | P-Coumaroyl-CoA, caffeoyl-CoA | (Legrand et al., 2016) |
| CiHQT2 | ANN12611.1 | Cichorium intybus | Quinate (<https://pubchem.ncbi.nlm.nih.gov/compound/Quinate>) | P-Coumaroyl-CoA, caffeoyl-CoA | (Legrand et al., 2016) |
| CiHQT3 | ANN12612.1 | Cichorium intybus | Quinate (<https://pubchem.ncbi.nlm.nih.gov/compound/Quinate>) | P-Coumaroyl-CoA, caffeoyl-CoA | (Legrand et al., 2016) |
| CiSHT1 | AXY93625.1 | Cichorium intybus | Putrescine (<https://pubchem.ncbi.nlm.nih.gov/substance/24278650>)  Spermidine (<https://pubchem.ncbi.nlm.nih.gov/compound/spermidine>)  Spermine (<https://pubchem.ncbi.nlm.nih.gov/compound/spermine>)  quinate (<https://pubchem.ncbi.nlm.nih.gov/compound/Quinate>)  shikimate (<https://pubchem.ncbi.nlm.nih.gov/compound/Shikimic-acid>) | Cinnamoyl-CoA,  caffeoyl-CoA,  feruloyl-CoA | (Delporte et al., 2018) |
| CiSHT2 | AXY93626.1 | Cichorium intybus | Putrescine (<https://pubchem.ncbi.nlm.nih.gov/substance/24278650>)  Spermidine (<https://pubchem.ncbi.nlm.nih.gov/compound/spermidine>)  Spermine (<https://pubchem.ncbi.nlm.nih.gov/compound/spermine>)  quinate (<https://pubchem.ncbi.nlm.nih.gov/compound/Quinate>)  shikimate (<https://pubchem.ncbi.nlm.nih.gov/compound/Shikimic-acid>) | Cinnamoyl-CoA,  caffeoyl-CoA,  feruloyl-CoA | (Delporte et al., 2018) |
| CmAAT1 | CAA94432.1 | Charentais melon | Hexanol (<https://pubchem.ncbi.nlm.nih.gov/substance/57650899>)  1-Butanol (<https://pubchem.ncbi.nlm.nih.gov/compound/1-butanol>)  Benzyl alcohol (<https://pubchem.ncbi.nlm.nih.gov/compound/Benzyl-alcohol>)  2-Phenylethanol (<https://pubchem.ncbi.nlm.nih.gov/compound/2-phenylethanol>)  1-Hexanol (<https://pubchem.ncbi.nlm.nih.gov/compound/1-Hexanol>)  Z-2-hexen-1-ol (<https://pubchem.ncbi.nlm.nih.gov/compound/2-Hexen-1-OL>) | Acetyl-CoA,  butanoyl-CoA | (Yahyaoui et al., 2002) |
| CrDAT | AAC99311.1 | Catharanthus roseus | Deacetylvindoline (<https://pubchem.ncbi.nlm.nih.gov/compound/Deacetylvindoline>) | Acetyl-CoA | (St-Pierre et al., 1998) |
| Dm3MAT1 | AAQ63615.1 | Dendranthema x morifolium | Cyanidin 3-O-glucoside (<https://pubchem.ncbi.nlm.nih.gov/compound/cyanidin-3-glucoside>)  Isoquercitrin (<https://pubchem.ncbi.nlm.nih.gov/compound/5484006>)  pelargonidin-3-O-beta-D-glucoside (<https://pubchem.ncbi.nlm.nih.gov/compound/Pelargonidin-3-O-beta-D-glucoside>)  delfinidin-3-O-glucoside (<https://pubchem.ncbi.nlm.nih.gov/compound/102515359>) | Malonyl-CoA,  succinyl-CoA,  cinyl-CoA,  methylmalonyl-CoA | (Suzuki et al., 2004b) |
| Dm3MAT2 | AAQ63616.1 | Dendranthema x morifolium | Cyanidin-3-(6'-Malonyl CoAonylglucoside) (<https://pubchem.ncbi.nlm.nih.gov/compound/Cyanidin-3-_6_-malonylglucoside>)  cyanidin 3-O-glucoside (<https://pubchem.ncbi.nlm.nih.gov/compound/cyanidin-3-glucoside>)  isoquercitrin (<https://pubchem.ncbi.nlm.nih.gov/compound/5484006>)  pelargonidin-3-O-beta-D-glucoside (<https://pubchem.ncbi.nlm.nih.gov/compound/Pelargonidin-3-O-beta-D-glucoside>)  delfinidin-3-O-glucoside (<https://pubchem.ncbi.nlm.nih.gov/compound/102515359>) | Malonyl-CoA,  succinyl-CoA,  cinyl-CoA,  methylmalonyl-CoA | (Suzuki et al., 2004b) |
| Dm3MAT3 | BAF50706 | Dendranthema x morifolium | Cyanidin 3-O-glucoside (<https://pubchem.ncbi.nlm.nih.gov/compound/cyanidin-3-glucoside>)  pelargonidin 3-O-glucoside (<https://pubchem.ncbi.nlm.nih.gov/compound/Kaempferol-7-o-D-glucopyranoside>)  delphinidin 3-O-glucoside (<https://pubchem.ncbi.nlm.nih.gov/compound/102515359>) | Malonyl-CoA,  succinyl-CoA | (Unno et al., 2007) |
| FaAAT | AAG13130.1 | Fragaria ananassa | benzol alcohol (<https://pubchem.ncbi.nlm.nih.gov/compound/Benzyl-alcohol>)  1-Butanol (<https://pubchem.ncbi.nlm.nih.gov/compound/1-butanol>)  Ethanol (<https://pubchem.ncbi.nlm.nih.gov/compound/702>) | Acetyl-CoA | (Unno et al., 2007) |
| Gt5AT | BAA74428.1 | Gentiana triflora | pelargonidin 3,5-diglucoside (<https://pubchem.ncbi.nlm.nih.gov/compound/Pelargonidin-3_5-diglucoside>)  cyanidin 3,5-O-diglucoside (<https://pubchem.ncbi.nlm.nih.gov/compound/Cyanidin-3_5-diglucoside>)  delphinidin 3,5-O-diglucoside (<https://pubchem.ncbi.nlm.nih.gov/compound/Delphinidin-3_5-diglucoside>) | Caffeoyl-CoA,  p-Coumaroyl-CoA | (Fujiwara et al., 1998) |
| DcHCBT | CAB06430.1 | Dianthus caryophyllus | Anthranilate (<https://pubchem.ncbi.nlm.nih.gov/compound/227>) | benzoyl-CoA,  cinnamoyl-CoA, coumaroyl-CoA | (Yang et al., 1997) |
| HmACT | BCN87077.1 | Hordeum vulgare | Agmatine (<https://pubchem.ncbi.nlm.nih.gov/compound/agmatine>)  Putrescine (<https://pubchem.ncbi.nlm.nih.gov/substance/24278650>)  Spermine (<https://pubchem.ncbi.nlm.nih.gov/compound/spermine>)  Spermidine (<https://pubchem.ncbi.nlm.nih.gov/compound/spermidine>) | Coumaroyl-CoA,  feruloyl-CoA,  caffeoyl-CoA | (Yamane et al., 2021) |
| LaHMT/HLT | BAD89275.1 | Lupinus albus | 13α-Hydroxylupanine (<https://pubchem.ncbi.nlm.nih.gov/compound/13-Hydroxylupanine>) | Tigloyl-CoA | (Okada et al., 2005) |
| HvACT | AAO73071.1 | Hordeum vulgare | Agmatine (<https://pubchem.ncbi.nlm.nih.gov/compound/agmatine>) | Coumaroyl-CoA,  feruloyl-CoA,  caffeoyl-CoA, | (Burhenne et al., 2003) |
| HvACT1-1 | BAF97626 .1 | Hordeum vulgare | Agmatine (<https://pubchem.ncbi.nlm.nih.gov/compound/agmatine>)  Putrescine (<https://pubchem.ncbi.nlm.nih.gov/substance/24278650>)  Spermine (<https://pubchem.ncbi.nlm.nih.gov/compound/spermine>)  Spermidine (<https://pubchem.ncbi.nlm.nih.gov/compound/spermidine>) | Coumaroyl-CoA,  feruloyl-CoA,  caffeoyl-CoA | (Yamane et al., 2021) |
| HvACT1-2 | BAF97627.1 | Hordeum vulgare | Agmatine (<https://pubchem.ncbi.nlm.nih.gov/compound/agmatine>)  Putrescine (<https://pubchem.ncbi.nlm.nih.gov/substance/24278650>)  Spermine (<https://pubchem.ncbi.nlm.nih.gov/compound/spermine>)  Spermidine (<https://pubchem.ncbi.nlm.nih.gov/compound/spermidine>) | Coumaroyl-CoA,  feruloyl-CoA,  caffeoyl-CoA | (Yamane et al., 2021) |
| MdAAT1 | AAU14879.2 | Malus domestica | 1-Butanol (<https://pubchem.ncbi.nlm.nih.gov/compound/1-butanol>)  eugenol (<https://pubchem.ncbi.nlm.nih.gov/compound/eugenol>)  1-Hexanol (<https://pubchem.ncbi.nlm.nih.gov/compound/1-Hexanol>)  2-Butanol (<https://pubchem.ncbi.nlm.nih.gov/compound/2-Butanol>)  chavicol (<https://pubchem.ncbi.nlm.nih.gov/compound/68148>)  isoeugenol (<https://pubchem.ncbi.nlm.nih.gov/compound/Isoeugenol>) | Acetyl-CoA,  butanoyl-CoA,  hexanoyl-CoA | (Yauk et al., 2017) |
| MtMaT1 | ABY91220.1 | Medicago truncatula | Naringenin 7-O-glucoside (<https://pubchem.ncbi.nlm.nih.gov/compound/Naringenin-7-O-glucoside_-tms> )  daidzin (<https://pubchem.ncbi.nlm.nih.gov/compound/Daidzin>)  naringin (<https://pubchem.ncbi.nlm.nih.gov/compound/Naringoside>)  formononetin 7-O-glucoside (<https://pubchem.ncbi.nlm.nih.gov/compound/442813>)  6,4-dihydroxy-7-O-glucosyl-isoflavone  Genistin (<https://pubchem.ncbi.nlm.nih.gov/compound/5281377>) | Malonyl- CoA | (Yu et al., 2008) |
| MtMaT2 | ABY91222.1 | Medicago truncatula | Naringenin 7-O-glucoside (<https://pubchem.ncbi.nlm.nih.gov/compound/Naringenin-7-O-glucoside_-tms>)  daidzin (<https://pubchem.ncbi.nlm.nih.gov/compound/Daidzin>)  naringin (<https://pubchem.ncbi.nlm.nih.gov/compound/Naringoside>)  formononetin 7-O-glucoside (<https://pubchem.ncbi.nlm.nih.gov/compound/442813>)  quercetin 3-O-glucoside (<https://pubchem.ncbi.nlm.nih.gov/compound/quercetin-3_-O-glucoside>)  6,4-dihydroxy-8-O-glucosyl-isoflavone  genistin (<https://pubchem.ncbi.nlm.nih.gov/compound/5281377>) | Malonyl- CoA | (Yu et al., 2008) |
| MtMaT3 | ABY91221.1 | Medicago truncatula | Naringenin 7-O-glucoside (<https://pubchem.ncbi.nlm.nih.gov/compound/Naringenin-7-O-glucoside_-tms>)  Daidzin (<https://pubchem.ncbi.nlm.nih.gov/compound/Daidzin>)  naringin (<https://pubchem.ncbi.nlm.nih.gov/compound/Naringoside>)  formononetin 7-O-glucoside (<https://pubchem.ncbi.nlm.nih.gov/compound/442813>)  quercetin 3-O-glucoside (<https://pubchem.ncbi.nlm.nih.gov/compound/quercetin-3_-O-glucoside>)  6,4-dihydroxy-7-O-glucosyl-isoflavone  Genistin (<https://pubchem.ncbi.nlm.nih.gov/compound/5281377>) | Malonyl- CoA | (Yu et al., 2008) |
| NaAT1 | AET80688.1 | Nicotiana attenuata | Putrescine (<https://pubchem.ncbi.nlm.nih.gov/substance/24278650>) | Coumaroyl-CoA,  caffeoyl-CoA,  feruloyl-CoA | (Onkokesung et al., 2011) |
| NaDH29 | AET80686.1 | Nicotiana attenuata | Spermidine (<https://pubchem.ncbi.nlm.nih.gov/compound/spermidine>) | Coumaroyl-CoA,  caffeoyl-CoA,  feruloyl-CoA | (Onkokesung et al., 2011) |
| NtHCT | CAD47830 .1 | Nicotiana tabacum | Shikimate (<https://pubchem.ncbi.nlm.nih.gov/compound/Shikimic-acid>)  quinate (<https://pubchem.ncbi.nlm.nih.gov/compound/Quinate>) | Coumaroyl-CoA,  caffeoyl-CoA,  feruloyl-CoA | (Hoffmann et al., 2003) |
| NtHQT | CAE46932.1 | Nicotiana tabacum | Quinate (<https://pubchem.ncbi.nlm.nih.gov/compound/Quinate>)  Shikimate (<https://pubchem.ncbi.nlm.nih.gov/compound/Shikimic-acid>) | Caffeoyl-CoA,  coumaroyl-CoA | (Niggeweg et al., 2004) |
| NtMAT1 | BAD93691.1 | Nicotiana tabacum | Kaempferol 3-O-glucoside (<https://pubchem.ncbi.nlm.nih.gov/compound/kaempferol-3-O-beta-D-glucoside_1>)  Quercetin 3-O-glucoside (<https://pubchem.ncbi.nlm.nih.gov/compound/Quercetin-3-beta-D-glucoside>)  7-Hydroxyflavone glucoside (<https://pubchem.ncbi.nlm.nih.gov/compound/7-Hydroxyflavon>) | Malonyl-CoA | (Taguchi et al., 2005) |
| OsPHT3 | NP_001390865.1 | Oryza sativa | Putrescine (<https://pubchem.ncbi.nlm.nih.gov/substance/24278650>) | P-Coumaroyl-CoA | (Fang et al., 2022) |
| OsPHT4 | XP_015651357.2 | Oryza sativa | Putrescine (<https://pubchem.ncbi.nlm.nih.gov/substance/24278650>) | P-Coumaroyl-CoA, feruloyl-CoA | (Fang et al., 2022) |
| OsPMT | NP_001403263.1 | Oryza sativa | Sinapyl alcohol (<https://pubchem.ncbi.nlm.nih.gov/compound/Sinapyl-alcohol>)  p-Coumaryl alcohol (<https://pubchem.ncbi.nlm.nih.gov/compound/p-Coumaryl-alcohol>) | P-Coumaroyl-CoA | (Withers et al., 2012) |
| OsTBT1 | ANQ47375.1 | Oryza sativa | Tryptamine (<https://pubchem.ncbi.nlm.nih.gov/compound/tryptamine>)  Serotonin (<https://pubchem.ncbi.nlm.nih.gov/compound/5202>)  tyramine (<https://pubchem.ncbi.nlm.nih.gov/compound/tyramine>) | Benzoyl-CoA,  coumaroyl-CoA,  caffeoyl-CoA | (Peng et al., 2016) |
| OsTBT2 | ANQ47376.1 | Oryza sativa | Tryptamine (<https://pubchem.ncbi.nlm.nih.gov/compound/tryptamine>)  serotonin (<https://pubchem.ncbi.nlm.nih.gov/compound/5202>)  tyramine (<https://pubchem.ncbi.nlm.nih.gov/compound/tyramine>) | Benzoyl-CoA | (Peng et al., 2016) |
| OsTHT1 | ANQ47373.1 | Oryza sativa | Tryptamine (<https://pubchem.ncbi.nlm.nih.gov/compound/tryptamine>)  tyramine (<https://pubchem.ncbi.nlm.nih.gov/compound/tyramine>)  Agmatine (<https://pubchem.ncbi.nlm.nih.gov/compound/agmatine>) | Benzoyl-CoA,  coumaroyl-CoA,  caffeoyl-CoA | (Peng et al., 2016) |
| OsTHT2 | ANQ47374.1 | Oryza sativa | Tryptamine (<https://pubchem.ncbi.nlm.nih.gov/compound/tryptamine>)  Serotonin (<https://pubchem.ncbi.nlm.nih.gov/compound/5202>)  tyramine (<https://pubchem.ncbi.nlm.nih.gov/compound/tyramine>) | Coumaroyl-CoA,  caffeoyl-CoA,  feruloyl-CoA | (Peng et al., 2016) |
| PhBPBT | AAU06226.1 | Petunia x hybrida | Benzoyl alcohol (<https://pubchem.ncbi.nlm.nih.gov/compound/244>)  2-Phenylethanol (<https://pubchem.ncbi.nlm.nih.gov/compound/2-phenylethanolv>) | Acetyl-CoA,  benzoyl-CoA | (Boatright et al., 2004) |
| PpAAT1 | QEZ90731.1 | Prunus persica | γ-Decalactone (<https://pubchem.ncbi.nlm.nih.gov/substance/329770261>) | 4-Hydroxydecanoyl-CoA | (Peng et al., 2020) |
| CaPun1 | AAV66311.1 | Capsaicin | vanillylamine (<https://pubchem.ncbi.nlm.nih.gov/compound/Vanillylamine>) | unknow | (Stewart Jr et al., 2005) |
| RhAAT1 | AAW31948.1 | Rosa hybrid cultivar | 1-Octanol (<https://pubchem.ncbi.nlm.nih.gov/compound/1-octanol>)  1-Hexanoyl (<https://pubchem.ncbi.nlm.nih.gov/compound/1-Hexanol>)  Citronellol (<https://pubchem.ncbi.nlm.nih.gov/substance/24893050>)  Geraniol (<https://pubchem.ncbi.nlm.nih.gov/compound/geraniol>) | Acetyl-CoA | (Shalit et al., 2003) |
| RsVs | CAD89104.2 | Rauvolfia serpentine | 16-epi-vellosimine (<https://pubchem.ncbi.nlm.nih.gov/compound/101348845>)  Gardneral | Acetyl-CoA | (Fan et al., 2016b) |
| SlAAT1 | NP_001234496.1 | Solanum lycopersicum | Benzyl alcohol (<https://pubchem.ncbi.nlm.nih.gov/compound/Benzyl-alcohol>)  1-Butanol (<https://pubchem.ncbi.nlm.nih.gov/compound/1-butanol>)  1-Pentanol (<https://pubchem.ncbi.nlm.nih.gov/compound/1-Pentanol>)  1-Butanol (<https://pubchem.ncbi.nlm.nih.gov/compound/1-butanol>)  1-Hexanol (<https://pubchem.ncbi.nlm.nih.gov/compound/1-Hexanol>)  2-methyl-1-butanol (<https://pubchem.ncbi.nlm.nih.gov/compound/2-methyl-1-butanol>) | Acetyl-CoA,  propinoyl-CoA,  butyryl-CoA | (Goulet et al., 2015) |
| SlASAT1 | ALU64003.1 | Solanum lycopersicum | Sucrose (<https://pubchem.ncbi.nlm.nih.gov/compound/sucrose>)  lactose (<https://pubchem.ncbi.nlm.nih.gov/compound/6134>)  cellobiose (<https://pubchem.ncbi.nlm.nih.gov/compound/cellobiose>)  trehalose (<https://pubchem.ncbi.nlm.nih.gov/compound/trehalose>) | iC5-CoA | (Fan et al., 2016a) |
| SlASAT2 | ALU64014.1 | Solanum lycopersicum | Acylsucrose S1:5(5) (<https://pubchem.ncbi.nlm.nih.gov/compound/122706451>) | aic5-CoA, nC12-CoA | (Fan et al., 2016a) |
| SlASAT3 | AJF98582.1 | Solanum lycopersicum | Acylsucrose S2:10(5, 5) (<https://pubchem.ncbi.nlm.nih.gov/substance/405233475>) | iC5-CoA | (Fan et al., 2016a) |
| SlASAT4 | AFM77971.1 | Solanum lycopersicum | Acylsucrose S3:22(5,5,12) (<https://pubchem.ncbi.nlm.nih.gov/substance/405233478>) | Acetyl-CoA | (Fan et al., 2016a) |
| SlHQT | CAE46933.1 | Solanum lycopersicum | Quinate (<https://pubchem.ncbi.nlm.nih.gov/compound/Quinate>) | Caffeoyl-CoA,  coumaroyl-CoA | (Moglia et al., 2014) |
| SlSHT | QNO39109.1 | Solanum lycopersicum | Spermidine (<https://pubchem.ncbi.nlm.nih.gov/compound/spermidine>) | P-Coumaroyl-CoA | (Perrin et al., 2021) |
| Ss5MAT1 | AAL50566.1 | Salvia splendens | Bisdemalonylsalvianin (<https://pubchem.ncbi.nlm.nih.gov/compound/Bisdemalonylsalvianin>)  monodemalonylsalvianin (<https://pubchem.ncbi.nlm.nih.gov/compound/5282162>) | Malonyl- CoA | (Suzuki et al., 2001) |
| Ss5MAT2 | AAR26385.1 | Salvia splendens | Monodemalonylsalvianin (<https://pubchem.ncbi.nlm.nih.gov/compound/5282162>) | Malonyl- CoA | (Suzuki et al., 2004c) |
| StFHT | ACS70946.1 | Solanum tuberosum | 1-Dodecanol (<https://pubchem.ncbi.nlm.nih.gov/compound/1-Dodecanol>)  1-Tetradecanol (<https://pubchem.ncbi.nlm.nih.gov/compound/1-tetradecanol>) | Feruloyl-CoA | (Serra et al., 2010) |
| TaACT1-1 | BCN87078.1 | Triticum aestivum | Agmatine (<https://pubchem.ncbi.nlm.nih.gov/compound/agmatine>)  Putrescine (<https://pubchem.ncbi.nlm.nih.gov/substance/24278650>)  Spermine (<https://pubchem.ncbi.nlm.nih.gov/compound/spermine>)  Spermidine (<https://pubchem.ncbi.nlm.nih.gov/compound/spermidine>) | Coumaroyl-CoA,  feruloyl-CoA,  caffeoyl-CoA | (Yamane et al., 2021) |
| TaACT1-2 | BCN87079.1 | Triticum aestivum | Agmatine (<https://pubchem.ncbi.nlm.nih.gov/compound/agmatine>)  Putrescine (<https://pubchem.ncbi.nlm.nih.gov/substance/24278650>)  Spermine (<https://pubchem.ncbi.nlm.nih.gov/compound/spermine>)  spermidine (<https://pubchem.ncbi.nlm.nih.gov/compound/spermidine>) | Coumaroyl-CoA,  feruloyl-CoA,  caffeoyl-CoA | (Yamane et al., 2021) |
| TcBAPT | AAL92459.1 | Taxus cuspidata | Baccatin III (<https://pubchem.ncbi.nlm.nih.gov/compound/Baccatin-III>) | β-Phenylalanoyl-CoA | (Walker et al., 2002a) |
| TcDBAT | AAF27621.1 | Taxus cuspidata | 10-DeAcetylbaccatin III (<https://pubchem.ncbi.nlm.nih.gov/compound/10-Deacetylbaccatin-III>) | Acetyl-CoA | (Walker et al, 2000a) |
| TcDBTNBT | AAM75818.1 | Taxus cuspidata | N-debenzoyl-CoAzoyl-(3'RS)-20 deoxytaxol (<https://pubchem.ncbi.nlm.nih.gov/compound/N-Debenzoyl-_3_-RS_-2_-deoxytaxol>) | Benzoyl-CoA | (Walker et al., 2002b) |
| TcTAT | AAF34254.1 | Taxus cuspidata | Taxa-4(20),11(12)-dien-5a-ol (<https://pubchem.ncbi.nlm.nih.gov/compound/Taxa-4_11-diene>) | Acetyl-CoA | (Walker et al., 2000) |
| TcTBT | AAG38049.1 | Taxus cuspidata | 2-Debenzoyl-7,13-diacetylbaccatin III (<https://pubchem.ncbi.nlm.nih.gov/compound/2-Debenzoyl-7_13-diacetylbaccatin-III>) | Benzoyl-CoA | (Walker et al, 2000b) |
| Vh3MAT1 | AAS77403.1 | Nicotiana tabacum | Quercetin 3-O-glucoside (<https://pubchem.ncbi.nlm.nih.gov/compound/quercetin-3_-O-glucoside>)  daidzein (<https://pubchem.ncbi.nlm.nih.gov/compound/daidzein>)  genistein (<https://pubchem.ncbi.nlm.nih.gov/compound/genistein>) | Malonyl-CoA,  acetyl-CoA, methylmalonyl-CoA, succinyl-CoA | (Suzuki et al., 2004a) |
| VlAMAT | AAW22989.1 | Vitis labrusca | 1-Butanol (<https://pubchem.ncbi.nlm.nih.gov/compound/1-butanol>) | Benzoyl-CoA, anthraniloyl-CoA,  acetyl-CoA | (Wang, 2005) |
| ZmGlossy2 | CAA61258.1 | Zea mays | C32 epicuticular waxes | unknow | (Tacke et al., 1995) |

The enzymes are selected biochemically characterized BAHDs to date.

Clicking the hyperlinks in Acceptor column will access to the PubMed database at NCBI-NIH to read the structure and annotation of each enzyme.

**References**

Beekwilder, J., Alvarez-Huerta, M., Neef, E., Verstappen, F.W.A., Bouwmeester, H.J., and Aharoni, A. (2004). Functional Characterization of Enzymes Forming Volatile Esters from Strawberry and Banana. *Plant physiol.* 135, 1865-1878.

Berger, A., Meinhard, J., and Petersen, M. (2006). Rosmarinic acid synthase is a new member of the superfamily of BAHD acyltransferases. *Planta.* 224, 1503-1510.

Boatright, J., Negre, F., Chen, X., Kish, C.M., Wood, B., Peel, G., et.al. (2004). Understanding in Vivo Benzenoid Metabolism in Petunia Petal Tissue. *Plant Physiol*. 135, 1993-2011.

Burhenne, K., Kristensen, B.K., and Rasmussen, S.K. (2003). A New Class of N-Hydroxycinnamoyltransferases: PURIFICATION, CLONING, AND EXPRESSION OF A BARLEY AGMATINE COUMAROYLTRANSFERASE (EC 2.3.1.64)*. *J Biol Chem*. 278, 13919-13927.

D’Auria, J.C. (2006). Acyltransferases in plants: a good time to be BAHD. *Curr. Plant Biol.* 9, 331-340.

D’Auria, J.C., Reichelt, M., Luck, K., Svatoš, A., and Gershenzon, J. (2007). Identification and characterization of the BAHD acyltransferase malonyl CoA: Anthocyanidin 5-O-glucoside-6″-O-malonyltransferase (At5MAT) in Arabidopsis thaliana. FEBS Letters. 581, 872-878.

Delporte, M., Bernard, G., Legrand, G., Hielscher, B., Lanoue, A., Molinié, R., et al. (2018). A BAHD neofunctionalization promotes tetrahydroxycinnamoyl spermine accumulation in the pollen coat of the Asteraceae family. *J. Exp. Bot.* 69, 5355-5371.

Eudes, A., Mouille, M., Robinson, D.S., Benites, V.T., Wang, G., Roux, L., et al. (2016). Exploiting members of the BAHD acyltransferase family to synthesize multiple hydroxycinnamate and benzoate conjugates in yeast. *Microb. Cell Factories.* 15, 198.

Fan, P., Miller, A.M., Schilmiller, A.L., Liu, X., Ofner, I., Jones, A.D.,et.al. (2016). In vitro reconstruction and analysis of evolutionary variation of the tomato acylsucrose metabolic network. *Proc Natl Acad Sci U S A.*113, E239-248.

Fang, H., Zhang, F., Zhang, C., Wang, D., Shen, S., He, F., et al. (2022). Function of hydroxycinnamoyl transferases for the biosynthesis of phenolamides in rice resistance to Magnaporthe oryzae. *J Genet Genomics* 49, 776-786.

Fujiwara, H., Tanaka, Y., Yonekura-Sakakibara, K., Fukuchi-Mizutani, M., Nakao, M., Fukui, Y., Yamaguchi, M., Ashikari, T., and Kusumi, T. (1998). cDNA cloning, gene expression and subcellular localization of anthocyanin 5-aromatic acyltransferase from Gentiana triflora. *Plant J.* 16, 421-431.

Gou, J.-Y., Yu, X.-H., and Liu, C.-J. (2009).  A hydroxycinnamoyltransferase responsible for synthesizing suberin aromatics in Arabidopsis. *Proc Natl Acad Sci U S A*. 106, 18855-18860.

Goulet, C., Kamiyoshihara, Y., Lam, Nghi B., Richard, T., Taylor, Mark G., Tieman, Denise M., and Klee, Harry J. (2015). Divergence in the Enzymatic Activities of a Tomato and Solanum pennellii Alcohol Acyltransferase Impacts Fruit Volatile Ester Composition. *Mol Plant.* 8, 153-162.

Grienenberger, E., Besseau, S., Geoffroy, P., Debayle, D., Heintz, D., Lapierre, C., et.al. (2009).  A BAHD acyltransferase is expressed in the tapetum of Arabidopsis anthers and is involved in the synthesis of hydroxycinnamoyl spermidines. *Plant J.* 58, 246-259.

Hoffmann, L., Maury, S., Martz, F., Geoffroy, P., and Legrand, M. (2003). Purification, Cloning, and Properties of an Acyltransferase Controlling Shikimate and Quinate Ester Intermediates in Phenylpropanoid Metabolism*. *J. Biol Chem.* 278, 95-103.

Hoffmann, L.A.C., Besseau, S., Geoffroy, P., Ritzenthaler, C.A., Meyer, D., Lapierre, C., et.al. (2005). Acyltransferase-catalysed p- coumarate ester formation is a committed step of lignin biosynthesis. *J Societa Botanica Italiana*. 139, 50 - 53.

Huang, A.C., Jiang, T., Liu, Y.-X., Bai, Y.-C., Reed, J., Qu, B., et.al. (2019). A specialized metabolic network selectively modulates *Arabidopsis* root microbiota. *Science.*364, eaau6389.

Irmisch, S., Jo, S., Roach, C.R., Jancsik, S., Man Saint Yuen, M., Madilao, L.L., O’Neil-Johnson, M., Williams, R., Withers, S.G., and Bohlmann, J. (2018). Discovery of UDP-Glycosyltransferases and BAHD-Acyltransferases Involved in the Biosynthesis of the Antidiabetic Plant Metabolite Montbretin A. *Plant cell*. 30, 1864-1886.

Kosma, D.K., Molina, I., Ohlrogge, J.B., and Pollard, M. (2012). Identification of an Arabidopsis Fatty Alcohol:Caffeoyl-Coenzyme A Acyltransferase Required for the Synthesis of Alkyl Hydroxycinnamates in Root Waxes1. *Plant Physiol.* 160, 237-248.

Legrand, G., Delporte, M., Khelifi, C., Harant, A., Vuylsteker, C., Mörchen, M., Hance, P., Hilbert, J.-L., and Gagneul, D. (2016). Identification and Characterization of Five BAHD Acyltransferases Involved in Hydroxycinnamoyl Ester Metabolism in Chicory. *Front Plant Sci.*;7:741.

Luo, J., Fuell, C., Parr, A., Hill, L., Bailey, P., Elliott, K., et.al. (2009). A novel polyamine acyltransferase responsible for the accumulation of spermidine conjugates in Arabidopsis seed. *Plant cell.* 21, 318-333.

Luo, J., Nishiyama, Y., Fuell, C., Taguchi, G., Elliott, K., Hill, L., et al. (2007). Convergent evolution in the BAHD family of acyl transferases: identification and characterization of anthocyanin acyl transferases from Arabidopsis thaliana. Plant J. 50, 678-695.

Moglia, A., Lanteri, S., Comino, C., Hill, L., Knevitt, D., Cagliero, C.,et.al. (2014). Dual Catalytic Activity of Hydroxycinnamoyl-Coenzyme A Quinate Transferase from Tomato Allows It to Moonlight in the Synthesis of Both Mono- and Dicaffeoylquinic Acids. *Plant Physiol.* 166, 1777-1787.

Molina, I., Li-Beisson, Y., Beisson, F., Ohlrogge, J.B., and Pollard, M. (2009). Identification of an Arabidopsis Feruloyl-Coenzyme A Transferase Required for Suberin Synthesis. *Plant physiol.* 151, 1317-1328.

Muroi, A., Ishihara, A., Tanaka, C., Ishizuka, A., Takabayashi, J., et.al. (2009). Accumulation of hydroxycinnamic acid amides induced by pathogen infection and identification of agmatine coumaroyltransferase in Arabidopsis thaliana. *Planta.* 230, 517-527.

Negruk, V., Yang, P., Subramanian, M., McNevin, J.P., and Lemieux, B. (1996). Molecular cloning and characterization of the CER2 gene of Arabidopsis thaliana. *Plant J*. 9(2):137-145.

Niggeweg, R., Michael, A.J., and Martin, C. (2004). Engineering plants with increased levels of the antioxidant chlorogenic acid. *Nat Biotechnol*. 22, 746-754.

Okada, T., Hirai, M.Y., Suzuki, H., Yamazaki, M., and Saito, K. (2005). Molecular Characterization of a Novel Quinolizidine Alkaloid O-Tigloyltransferase: cDNA Cloning, Catalytic Activity of Recombinant Protein and Expression Analysis in Lupinus Plants. *Plant Cell Physiol*. 46, 233-244.

Onkokesung, N., Gaquerel, E., Kotkar, H., Kaur, H., Baldwin, I.T., and Galis, I. (2011). MYB8 Controls Inducible Phenolamide Levels by Activating Three Novel Hydroxycinnamoyl-Coenzyme A:Polyamine Transferases in Nicotiana attenuata. *Plant physiol.* 158, 389-407.

Peng, B., Yu, M., Zhang, B., Xu, J., and Ma, R. (2020). Differences in PpAAT1 Activity in High- and Low-Aroma Peach Varieties Affect γ-Decalactone Production1. *Plant physiol.* 182, 2065-2080.

Peng, M., Gao, Y., Chen, W., Wang, W., Shen, S., Shi, J., et al. (2016). Evolutionarily Distinct BAHD N-Acyltransferases Are Responsible for Natural Variation of Aromatic Amine Conjugates in Rice. *Plant cell.* 28, 1533-1550.

Perrin, J., Kulagina, N., Unlubayir, M., Munsch, T., Carqueijeiro, I., Dugé de Bernonville, T., et al. (2021). Exploiting Spermidine N -Hydroxycinnamoyltransferase Diversity and Substrate Promiscuity to Produce Various Trihydroxycinnamoyl Spermidines and Analogues in Engineered Yeast. *ACS Synth Biol*. 10, 286-296.

Rautengarten, C., Ebert, B., Ouellet, M., Nafisi, M., Baidoo, E.E., Benke, P., et al. (2012). Arabidopsis Deficient in Cutin Ferulate encodes a transferase required for feruloylation of ω-hydroxy fatty acids in cutin polyester. *Plant physiol*. 158, 654-665.

Serra, O., Hohn, C., Franke, R., Prat, S., Molinas, M., and Figueras, M. (2010). A feruloyl transferase involved in the biosynthesis of suberin and suberin-associated wax is required for maturation and sealing properties of potato periderm. *Plant J.* 62, 277-290.

Shalit, M., Guterman, I., Volpin, H., Bar, E., Tamari, T., Menda, N., et al. (2003). Volatile Ester Formation in Roses. Identification of an Acetyl-Coenzyme A. Geraniol/Citronellol Acetyltransferase in Developing Rose Petals. *Plant physiol.* 131, 1868-1876.

Sonnante, G., D'Amore, R., Blanco, E., Pierri, C.L., De Palma, M., Luo, J., et.al. (2010). Novel Hydroxycinnamoyl-Coenzyme A Quinate Transferase Genes from Artichoke Are Involved in the Synthesis of Chlorogenic Acid. *Plant Physiol.* 153, 1224-1238.

St-Pierre, B., Laflamme, P., Alarco, A.-M., D, V., and Luca, E. (1998). The terminal O-acetyltransferase involved in vindoline biosynthesis defines a new class of proteins responsible for coenzyme A-dependent acyl transfer. *Plant J*. 14, 703-713.

Stewart Jr, C., Kang, B.-C., Liu, K., Mazourek, M., Moore, S.L., et.al. (2005). The Pun1 gene for pungency in pepper encodes a putative acyltransferase. *Plant J*. 42, 675-688.

Suzuki, H., Nakayama, T., Nagae, S., Yamaguchi, M.-A., Iwashita, T., Fukui, Y., et.al. (2004a). cDNA cloning and functional characterization of flavonol 3-O-glucoside-6″-O-malonyltransferases from flowers of Verbena hybrida and Lamium purpureum. J. Mol. Catal*.* 28, 87-93.

Suzuki, H., Nakayama, T., Yamaguchi, M.-a., and Nishino, T. (2004b). cDNA cloning and characterization of two Dendranthema×morifolium anthocyanin malonyltransferases with different functional activities. *Plant Sci.* 166, 89-96.

Suzuki, H., Nakayama, T., Yonekura-Sakakibara, K., Fukui, Y., Nakamura, N., Nakao, M., et.al. (2001). Malonyl-CoA:Anthocyanin 5-O-Glucoside-6‴-O-Malonyltransferase from Scarlet Sage (Salvia splendens) Flowers: ENZYME PURIFICATION, GENE CLONING, EXPRESSION, AND CHARACTERIZATION*210. *J. Biol Chem.* 276, 49013-49019.

Suzuki, H., Sawada, S.y., Watanabe, K., Nagae, S., Yamaguchi, M.-a., Nakayama, T., et.al. (2004c). Identification and characterization of a novel anthocyanin malonyltransferase from scarlet sage (Salvia splendens) flowers: an enzyme that is phylogenetically separated from other anthocyanin acyltransferases. *J. Biol Chem* 38, 994-1003.

Tacke, E., Korfhage, C., Michel, D., Maddaloni, M., Motto, M., Lanzini, S., et.al. (1995). Transposon tagging of the maize Glossy2 locus with the transposable element En/Spm. *Plant J*. *8*, 907-917.

Taguchi, G., Shitchi, Y., Shirasawa, S., Yamamoto, H., and Hayashida, N. (2005). Molecular cloning, characterization, and downregulation of an acyltransferase that catalyzes the malonylation of flavonoid and naphthol glucosides in tobacco cells. *Plant J*.42, 481-491.

Taguchi, G., Ubukata, T., Nozue, H., Kobayashi, Y., Takahi, M., Yamamoto, H., and Hayashida, N. (2010). Malonylation is a key reaction in the metabolism of xenobiotic phenolic glucosides in Arabidopsis and tobacco. *Plant J*. 63, 1031-1041.

Torrens-Spence, M.P., Bobokalonova, A., Carballo, V., Glinkerman, C.M., Pluskal, T., Shen, A., and Weng, J.-K. (2019). PBS3 and EPS1 Complete Salicylic Acid Biosynthesis from Isochorismate in Arabidopsis. *Mol Plant.*12, 1577-1586.

Unno, H., Ichimaida, F., Suzuki, H., Takahashi, S., Tanaka, Y., Saito, A., et.al. (2007). Structural and Mutational Studies of Anthocyanin Malonyltransferases Establish the Features of BAHD Enzyme Catalysis*. *J Biol Chem.* 282, 15812-15822.

Walker, K., and Croteau, R. (2000a). Molecular cloning of a 10-deacetylbaccatin III-10-O-acetyl transferase cDNA from Taxus and functional expression in Escherichia coli. *Proc Natl Acad Sci U S A*. 97, 583-587.

Withers, S., Lu, F., Kim, H., Zhu, Y., Ralph, J., and Wilkerson, C.G. (2012). Identification of Grass-specific Enzyme That Acylates Monolignols with p-Coumarate*. *J Biol Chem.* 287, 8347-8355.

Yahyaoui, F.E.L., Wongs-Aree, C., Latché, A., Hackett, R., Grierson, D., and Pech, J.-C. (2002). Molecular and biochemical characteristics of a gene encoding an alcohol acyl-transferase involved in the generation of aroma volatile esters during melon ripening. *Eur J Biochem.* 269, 2359-2366.

Yamane, M., Takenoya, M., Yajima, S., and Sue, M. (2021). Molecular and structural characterization of agmatine coumaroyltransferase in Triticeae, the key regulator of hydroxycinnamic acid amide accumulation. *Phytochemistry.*189,112825.

Yan, X., Qin, X., Li, W., Liang, D., Qiao, J., and Li, Y. (2020). Functional characterization and catalytic activity improvement of BAHD acyltransferase from Celastrus angulatus Maxim. *Planta*. 252, 6.

Yang, Q., Reinhard, K., Schiltz, E., and Matern, U. (1997). Characterization and heterologous expression of hydroxycinnamoyl/benzoyl-CoA:anthranilate N-hydroxycinnamoyl/benzoyltransferase from elicited cell cultures of carnation, Dianthus caryophyllus L. *Plant Mol Biol*. 35, 777-789.

Yauk, Y.-K., Souleyre, E.J.F., Matich, A.J., Chen, X., Wang, M.Y., Plunkett, B., et al. (2017). Alcohol acyl transferase 1 links two distinct volatile pathways that produce esters and phenylpropenes in apple fruit. Plant J.91, 292-305.

Yu, X.-H., Chen, M.-H., and Liu, C.-J. (2008). Nucleocytoplasmic-localized acyltransferases catalyze the malonylation of 7-O-glycosidic (iso)flavones in Medicago truncatula. *Plant J*. 55, 382-396.
